# Systems Chronotherapeutics

**DOI:** 10.1124/pr.116.013441

**Published:** 2017-04

**Authors:** Annabelle Ballesta, Pasquale F. Innominato, Robert Dallmann, David A. Rand, Francis A. Lévi

**Affiliations:** Warwick Medical School (A.B., P.F.I., R.D., F.A.L.) and Warwick Mathematics Institute (A.B., D.A.R.), University of Warwick, Coventry, United Kingdom; Warwick Systems Biology and Infectious Disease Epidemiological Research Centre, Senate House, Coventry, United Kingdom (A.B., P.F.I., R.D., D.A.R., F.A.L.); INSERM–Warwick European Associated Laboratory “Personalising Cancer Chronotherapy through Systems Medicine” (C2SysMed), Unité mixte de Recherche Scientifique 935, Centre National de Recherche Scientifique Campus, Villejuif, France (A.B., P.F.I., R.D., D.A.R., F.A.L.); and Queen Elisabeth Hospital Birmingham, University Hospitals Birmingham National Health Service Foundation Trust, Cancer Unit, Edgbaston Birmingham, United Kingdom (P.F.I., F.A.L.)

## Abstract

Chronotherapeutics aim at treating illnesses according to the endogenous biologic rhythms, which moderate xenobiotic metabolism and cellular drug response. The molecular clocks present in individual cells involve approximately fifteen clock genes interconnected in regulatory feedback loops. They are coordinated by the suprachiasmatic nuclei, a hypothalamic pacemaker, which also adjusts the circadian rhythms to environmental cycles. As a result, many mechanisms of diseases and drug effects are controlled by the circadian timing system. Thus, the tolerability of nearly 500 medications varies by up to fivefold according to circadian scheduling, both in experimental models and/or patients. Moreover, treatment itself disrupted, maintained, or improved the circadian timing system as a function of drug timing. Improved patient outcomes on circadian-based treatments (chronotherapy) have been demonstrated in randomized clinical trials, especially for cancer and inflammatory diseases. However, recent technological advances have highlighted large interpatient differences in circadian functions resulting in significant variability in chronotherapy response. Such findings advocate for the advancement of personalized chronotherapeutics through interdisciplinary systems approaches. Thus, the combination of mathematical, statistical, technological, experimental, and clinical expertise is now shaping the development of dedicated devices and diagnostic and delivery algorithms enabling treatment individualization. In particular, multiscale systems chronopharmacology approaches currently combine mathematical modeling based on cellular and whole-body physiology to preclinical and clinical investigations toward the design of patient-tailored chronotherapies. We review recent systems research works aiming to the individualization of disease treatment, with emphasis on both cancer management and circadian timing system–resetting strategies for improving chronic disease control and patient outcomes.

## Graphical abstract

**Figure:**
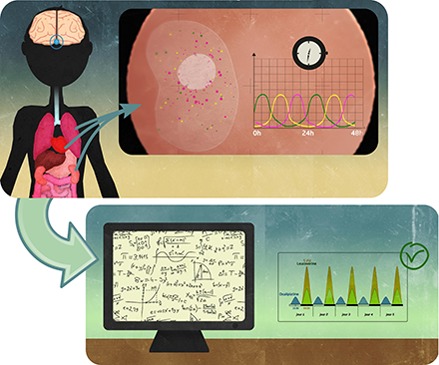

## I. Introduction: Systems Approaches to Optimize Chronotherapeutics

Broad interpatient variability in diseases and response to treatments has become increasingly apparent, so that personalizing medicine appears to be needed to ensure maximum treatment efficacy and minimum unwanted toxicities. However, a recent appraisal cautions the lack of consistent clinical benefits using the current personalized medicine concepts (Tannock and Hickman, 2016). Optimizing therapeutic strategies should hence encompass both the specificities of the patient’s pathology and the patient’s genetics and lifestyle (Khera et al., 2016; Califano and Alvarez, 2017). To this end, multitype and multiscale datasets have been generated in preclinical studies in cell cultures and in laboratory animals, and in clinical investigations involving populations of patients or individual subjects (Alvarez et al., 2016). The large volumes of data that are thus generated across species require dedicated approaches to properly analyze each individual dataset, to handle the complexity arising from multiple data types and dimensions, and to finally translate the results into individualized therapies. The rise of genomics and the accumulation of large amounts of data and longitudinal and dense multidimensional time series have paved the way for a new systems-based approach to biology. Systems approaches are defined in this work as interdisciplinary methodologies combining mathematical, statistical, technological, experimental, and clinical expertise for the development of dedicated devices, theoretical algorithms, and clinical protocols enabling treatment individualization.

Systems medicine involves the implementation of such systems biology approaches in medical concepts, research, and practice, through iterative and reciprocal feedback between clinical investigations and practice and computational, statistical, and mathematical analysis, as it has been emphasized in the Roadmap of the Coordinated Action for Systems Medicine (CaSyM) from the European Union (https://www.casym.eu), and other consortia (Anderson and Quaranta, 2008; Agur et al., 2014; Wolkenhauer et al., 2014; Iyengar et al., 2015). The aim is a novel appraisal of pathogenetic mechanisms, disease progression and remission, disease spread and cure, treatment responses and adverse events, as well as disease prevention both at the epidemiologic and individual patient level (CaSyM, 2014). Indeed, systems medicine aims at a measurable improvement of patient health through systems-based approaches and practice, which will enable a more predictive, personalized, participatory, and preventive (P4) medicine (Hood and Friend, 2011; Boissel et al., 2015).

Many rhythms have been found in all living beings, with periods ranging from milliseconds to years (Halberg, 1969). Although endogenicity characterizes biologic rhythms irrespective of period length, the molecular mechanisms at work can vary largely among the several kinds of biologic oscillators that reside in cells, tissues, organs, and whole organisms (Goldbeter et al., 2010). Systems chronotherapeutics aim at encompassing this underlying complex system and its dynamics toward the optimization of circadian-based treatment on patient-specific bases. To this end, experimental, translational, clinical, and multiscale modeling investigations have jointly aimed at representing the circadian control in healthy organs involved in drug pharmacology (e.g., hepatic metabolism, renal clearance) and/or most susceptible to being injured, as well as in diseased tissues (Bass and Lazar, 2016; Mermet et al., 2016; Panda, 2016).

In this study, we first describe the mammalian circadian timing system (CTS) and recent methods to longitudinally assess it at multiple levels in cell culture, laboratory animals, and individual patients, as a prerequisite for multiscale theoretical approaches. Then we provide the current state of the art of preclinical and clinical chronotherapeutics, and available technologies for chronomodulated drug administration. Next, we review recent systems approaches to optimize and personalize chronotherapeutics and show their relevance for improving cancer therapy. The potential of systems chronotherapeutics is further illustrated for cardiovascular, metabolic, and inflammatory disorders. The issue of CTS disruption is then addressed, regarding its clinical impact and the theoretical methods that could help design clocks resetting and synchronizing strategies. Finally, we discuss the current challenges toward a translation of systems approaches into the clinics.

## II. The Circadian Timing System and Its Multilevel Intersubject Variabilities

**Fig. 1.**
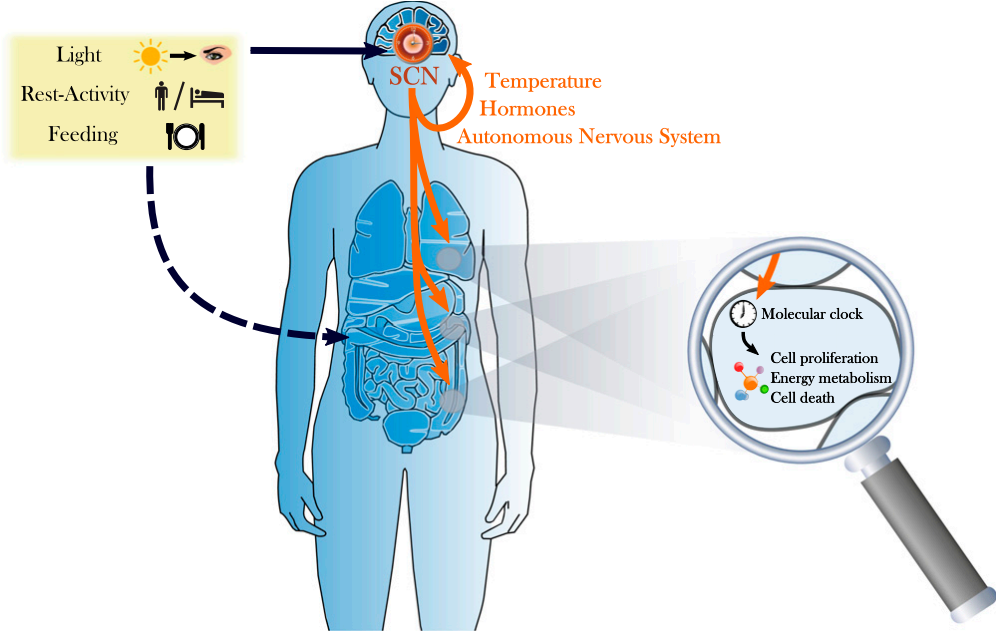
The CTS. The CTS is composed of a central pacemaker located in the SCN that displays autonomous circadian oscillations, but is also entrained by external cues such as light or socioprofessional activities. The SCN further generate rhythmic physiologic signals exerting a control on the autonomous molecular clocks present in each nucleated cell, which, in turn, induce oscillations in the expression of a large number of genes involved in key intracellular processes.

Timekeeping systems can be found in the vast majority of organisms on Earth, and they are believed to confer a selective advantage (Woelfle et al., 2004; Spoelstra et al., 2016). These biologic clocks most likely have evolved to anticipate recurrent daily changes in environmental conditions caused by the earth’s rotation around its own axis. With a period of about 24 hours (Latin: circa = about, dies = day), endogenous circadian clocks prepare organisms for important daily events such as the availability of food or changes in environmental temperature by orchestrating behavior and physiology before these events occur. In mammals, many biologic functions are modulated by the CTS as a consequence of endogenous temporal regulations at various levels influenced by external cues (Fig. 1).

### A. Structure of the CTS

#### 1. The Whole-Hierarchical Organization

The CTS is the entirety of all oscillators in an organism and their coupling through various physiologic processes. However, not all clocks are equal. The central or master pacemaker of the CTS resides in the suprachiasmatic nuclei (SCN) located in the ventral hypothalamus. They display endogenous circadian oscillations both at the cell and tissue levels and in their outputs toward other organs. Ablation of the SCN leads to arrhythmic behavior, physiology, and hormonal secretions (Moore and Eichler, 1972; Stephan and Zucker, 1972). Various mechanisms for how the circadian information is then transmitted to the rest of the body have been elucidated. The SCN exert a control on the organism through the rhythmic regulation of physiologic processes, including temperature, hormonal levels, and/or the autonomous nervous system (Lévi and Schibler, 2007). Melatonin, a hormone that is released by the pineal gland, is one example of a multisynaptic output of the SCN (Tordjman et al., 2017). It is widely used as a phase marker for the SCN, and, interestingly, its secretion has been shown to feedback on the SCN (Shimomura et al., 2010). At the peripheral level, each nucleated cell is endowed with a molecular circadian clock that generates autonomous intracellular circadian variations and is under the control of both SCN-driven systemic and tissue-level factors. In physiologic conditions, the oscillators in this complex interacting system display stable and well-defined phase relationships with each other. In fact, multiorgan high-density time course experiments in mice have revealed that at least the core clock genes exhibit similar phases throughout at least a dozen different tissues (Zhang et al., 2014b). Although a growing number of pathways for resetting cellular clocks are discovered, it is largely unknown how all of these interact in vivo and how the various tissues maintain coherent phase relationships with each other and with the environmental cycles. A number of possible mechanisms have already been proposed, and most likely all of these signals contribute in a tissue-specific manner (Schibler et al., 2015).

To be useful for anticipating environmental changes or specific events, the organism has to synchronize its clocks with outside cues. There are various of these so-called Zeitgeber (German for “time givers”) or synchronizers that entrain the CTS components to a robust 24-hour rhythm by resetting the phase or influencing the amplitude of the CTS oscillators at the cellular as well as organismal level (Fig. 1). One of the most well-studied Zeitgeber is the alternation of light and darkness over 24 hours. In mammals, light is exclusively detected by the retina through classic photoreceptors as well as specialized retinal ganglion cells that have direct monosynaptic connections into the SCN (Peirson et al., 2005). Rest-activity patterns, including sleep-wake cycles, familial and professional interactions, and physical exercise, also influence the central clock, whereas meal timing impacts metabolism-linked peripheral clocks.

#### 2. The Parts—Cellular Circadian Clocks

Under physiologic conditions, presumably all mammalian cells in the body possess a functional circadian oscillator. In principle, the underlying molecular mechanism is a negative feedback loop (Jolley et al., 2012). This basic mechanism was first described in fruit flies (Hardin et al., 1990). Although the players vary, the basic building principle is conserved across phyla and can be found in temporal variations in unicellular cyanobacteria as well as mammalian cells (Brown et al., 2012). Interestingly, the clock genes that contribute to the core transcriptional/(post-)translational feedback loops have largely been found using forward genetic N-ethyl-N-nitrosourea mutagenesis screens for dominant mutations or targeted transgenics on homologs of known clock genes in other organisms. Deletion or mutation of most of these genes leads to strongly disrupted circadian rhythms in behavior.

In mammals, the core clock genes of these oscillators are known in great detail, and the mechanism is briefly described below (Takahashi, 2017). The transcriptional activator complex of BMAL1 and its partner CLOCK or NPAS2 binds to short palindromic so-called E-box elements in the promoter of *PER* and *CRY* repressor genes (DeBruyne et al., 2007). After translation and post-transcriptional modifications, PER and CRY proteins re-enter the nucleus as a complex and switch off their own transcription, thereby closing the feedback loop (Padmanabhan et al., 2012). After about 24 hours, the repressor complex is removed and a new activation cycle can begin. Interlocked with this first discovered loop is a secondary or stabilizing loop. Driven by transcriptional activation through BMAL1-containing complexes, this loop is closed by direct REV-ERB and retinoic acid–related orphan nuclear receptor feedback on the transcriptional activity of *BMAL1* through retinoic acid–related orphan nuclear receptor elements in the *BMAL1* promoter (Guillaumond et al., 2005). The period, amplitude, and phase of this oscillator are largely determined by post-transcriptional modifications influencing nuclear transport or degradation of the repressor complexes as for casein kinase (CK)1 and E3 ligase activity of F-box proteins on PERs and CRYs (Busino et al., 2007; Meng et al., 2008; Etchegaray et al., 2009; Yoo et al., 2013).

Sumoylation (Cardone et al., 2005), acetylation (Doi et al., 2006; Hirayama et al., 2007; Asher et al., 2008), dephosphorylation (Reischl and Kramer, 2011), and ubiquitination (DeBruyne et al., 2015) further post-translationally regulate the clock proteins (Hirano et al., 2016). Furthermore, epigenetic regulation has been found to play a role in clock regulation (Papazyan et al., 2016) and rhythmic changes in the chromatin landscape of the DNA (Koike et al., 2012) and histone and DNA modifications have been reported (Ripperger and Merrow, 2011; Sahar and Sassone-Corsi, 2013; Azzi et al., 2014), and so have splicing and RNA modification as well as ribosomal translation (McGlincy et al., 2012; Lim and Allada, 2013; Perez-Santangelo et al., 2014; Jang et al., 2015; Janich et al., 2015).

All of these different layers of circadian regulation not only drive the core clock mechanism but, importantly, modulate many downstream processes (Chaix et al., 2016). In fact, up to 40% of the transcriptome is thought to oscillate with circadian period, and a similar proportion of 20**–**40% of the proteome and metabolome has also been found to exhibit circadian patterns (Dallmann et al., 2012; Davies et al., 2014; Mauvoisin et al., 2014; Robles et al., 2014; Zhang et al., 2014a; Giskeodegard et al., 2015).

Importantly, this core clock mechanism is linked to cellular functions on many levels. Although probably more than one of the above-mentioned mechanisms is responsible for this large proportion of rhythmic features, E-boxes are probably the simplest and most immediate way to control gene expression. It has to be noted, however, that the phase of many circadian genes is not only regulated by BMAL1:CLOCK binding to the E-box but also various other transcription factors that might change the phase of transcription possibly conveying tissue-specific regulation (Menet et al., 2012). In addition, further mechanisms have been discovered to tune phase and amplitude of clock-controlled genes in peripheral tissues. microRNA tissue-specific regulation of gene expression phase and amplitude was found (Du et al., 2014). Some of these genes are transcription factors, too, and control themselves as well as further sets of genes with a different phase compared with E-box**–**driven rhythmic genes (Bozek et al., 2007). The intricate relationship of the circadian oscillator with another highly controlled and rhythmic process, that is, the cell cycle, is discussed in detail below. Of course, this also has implications for apoptosis and the signaling pathways involved in its induction (Lee and Sancar, 2011a,b).

### B. Multiscale Circadian Assessment Enables Systems Approaches

**Fig. 2.**
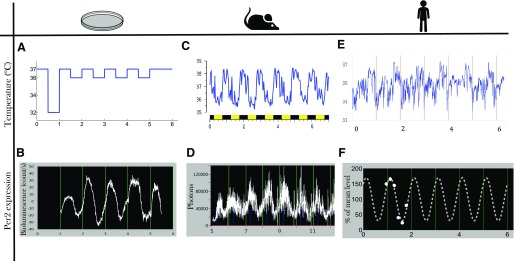
Multiscale longitudinal assessment of the CTS. Recent technologies allow for the recording of biomarkers of the CTS at multiple scales: in Per2::luc Hepa1-6 cell culture, imposed temperature cycles (A) and Per2 bioluminescence measured by Lumicycle (B); in individual B6D2F1 male mice entrained in LD12:12, body temperature recorded by telemetry (C); and, in Per2::luc animals, bioluminescence recorded in RT-Bio (D); in individual young male healthy volunteers, skin temperature recorded though new thoracic wearable sensors (E, In Casa project), and individual Per2 mRNA level in peripheral blood mononuclear cells (F, dots are data from Teboul et al., 2005; dotted line is the best-fit cosinor model). Longitudinal measurement over several days of molecular biomarkers is currently not available in the clinics, and multiscale systems approaches aim at predicting from preclinical results and clinical investigations the patient-specific dynamical information needed for treatment personalization. Time is expressed in days. Zero represents midnight (clock hours) on the first day of experiment.

Systems approaches were enabled by the recent development of experimental and clinical technologies allowing for longitudinal continuous measurements over several circadian periods of various components of the CTS in a single cell, a population of cells, as well as a laboratory animal, or a human subject in a nonrestrained environment (Fig. 2).

#### 1. Preclinical Longitudinal Circadian Studies toward Systems Approaches

The rapid progress made in cell culture synchronization methods, bioluminescence/fluorescence reporter technology, and dedicated dynamic imaging developments has recently fostered systems chronobiology and chronopharmacology studies. In particular, the use of real-time reporters allows for a quantitative definition of circadian period, phase, and amplitude of oscillations and thus can help in uncovering even subtle phenotypes (e.g., Duong et al., 2011). The cell transfection of multiple reporters and its single-cell imaging have further enabled the experimental investigation of the control of the molecular circadian clock on intracellular pathways involved in drug response (Feillet et al., 2014). Interestingly for the systems approach are the multiscale applications of such technologies that link mechanistic insights to the molecular clock in single cells or cell populations to whole organism dynamical behaviors. Indeed, real-time recording of liver gene expressions in freely moving individual mice has recently been enabled by the development of new devices (Saini et al., 2013). These technologies also called for the design of dedicated statistical signal-processing methods (Costa et al., 2013). Furthermore, with the availability of various “omics” techniques, systems approaches to further elucidate the circadian clock mechanism have been attempted. Of course, big data have necessitated new data mining strategies, and machine-learning algorithms have contributed to more accurately define the molecular clockwork (e.g., Anafi et al., 2014).

#### 2. Longitudinal Assessment of the Human CTS toward Personalized Medicine

Personalization systems approaches would not be possible without the recent development of technologies dedicated to assess circadian rhythms in individual human subjects. The patient’s CTS is either assessed through wearable sensors recording continuous data for several days, or through repeated measures, mainly in the blood, the saliva, the breath, or the urine as multiple sampling of any other human tissues would often be unpractical and/or unethical. To properly evaluate circadian function, it is required to identify the most pertinent circadian biomarkers and to design devices monitoring them over several days or weeks with the least discomfort for the patient. Generally, the most relevant biomarker rhythms are constituted by those that act also as resetting cues for the molecular peripheral clocks, or that also signal the central pacemaker (Innominato et al., 2014).

The rest-activity rhythm is the most widely assessed in patients, because its pattern can be easily evaluated using a small triaxial accelerometer (the actigraph), worn most often on the wrist, but also on the arm, thorax, hip, or ankle (Ancoli-Israel et al., 2003). Longitudinal wrist-actigraphy monitoring in individual cancer patients has indeed demonstrated its validity for prognosis prediction and for association with patient-reported outcome measures (Mormont and Waterhouse, 2002; Payne, 2011; Lévi et al., 2014). Actigraphy is particularly useful as it allows assessment of both locomotor activity and sleep, which are relevant biomarkers for many diseases (Broderick et al., 2014; Madsen et al., 2015).

More recently, temperature rhythm measured through thermal patches on the proximal (i.e., thoracic) or distal (i.e., forearm) skin has demonstrated its relevance in cancer patients (Roche et al., 2014; Ortiz-Tudela et al., 2016). A new device simultaneously recording rest-activity, position, and thoracic skin temperature was tested in the pilot clinical study Picado within a Domomedicine platform also monitoring patient daily weight variations and self-assessed quality of life and symptoms (Maurice et al., 2015).

Wearable technologies have also been developed to assess ECG and heart rate variability, as a surrogate of autonomic nervous system balance in the short- or medium-term (Melillo et al., 2015). Indeed, this biomarker has shown clinical value in hypertensive patients to predict fallers (Melillo et al., 2015) and in cancer patients (Palesh et al., 2008; Giese-Davis et al., 2015).

One of the most extensively assessed circadian rhythms in patients is cortisol, an adrenal hormone that can be easily measured in saliva as well as in blood. Such rhythm had an independent prognostic role in patients with breast, ovarian, kidney, or lung, but not colorectal cancer (Sephton et al., 2000, 2013). However, the vast majority of cortisol time series in patients only involve diurnal samples, with few, if any, night samples. To address this issue, a new sensor was developed that allows for the continuous measurement over 3 consecutive days of cortisol in the skin interstitial fluid, where cortisol levels are actually higher than in the saliva (Venugopal et al., 2011). Melatonin rhythm can also be measured in the saliva to assess the amplitude and phase of the central pacemaker with implication in various diseases, including brain injuries or sleep-wake disorders (Sletten et al., 2010; Burgess et al., 2015; Grima et al., 2016). Next, the least invasive way to monitor the transcriptional output of the molecular clock is to measure the expression profiles of core clock genes in peripheral blood mononuclear cells (Fig. 2). This approach is feasible in healthy subjects (Boivin et al., 2003; Takimoto et al., 2005), albeit atypical patterns can be found (Teboul et al., 2005), and only scarce data are available in patients. Finally, plasma, saliva, or even breath circadian “omics” datasets such as transcriptome and metabolome are now available in humans (Dallmann et al., 2012; Martinez-Lozano Sinues et al., 2014).

### C. Multilevel Intersubject Variability in the Human CTS Advocating for Personalized Chronotherapeutics

The timing of several circadian rhythms can vary among individuals with respect to sex, age, genetic background, and lifestyle (Ortiz-Tudela et al., 2013). Epidemiologic large-scale studies using the Munich Chronotype Questionnaire in more than 55,000 human subjects revealed large variation in behaviors within the 24-hour span (Roenneberg et al., 2007). Wrist actigraphy has further uncovered large intersubject differences in circadian phase and amplitude in a pooled analysis involving 436 patients with metastatic colorectal cancer. For instance, the circadian maximum in the activity of these patients was spread over a 10-hour span (Innominato et al., 2014). Moreover, interpatient differences of up to 12 hours were found in the circadian acrophase of skin surface temperature rhythms in 24 metastatic gastrointestinal cancer patients (Roche et al., 2014; Ortiz-Tudela et al., 2016).

Strong experimental and clinical evidence suggests that these interindividual differences in circadian physiology might translate at the molecular clock level (Cermakian and Boivin, 2003). The phase and amplitude of mRNA levels of *PER2*, *BMAL1*, and *REV*-*ERBα* expression measured over 24 hours through repeated sampling of peripheral blood cells of healthy young male subjects greatly varied among subjects, although this was not captured in wrist activity (Teboul et al., 2005). New real-time reporter techniques have also been instrumental to further discover significant variations in the in vitro circadian period of human fibroblasts taken from healthy individuals as a surrogate for in vivo human diurnal preferences (Brown et al., 2008; Pagani et al., 2010). Furthermore, the circadian expression of nearly 2000 genes in the oral mucosa differed between healthy male and female human subjects (Bjarnason et al., 2001). Moreover, findings showing that allelic variation of clock genes can influence the individual timing of cellular responses to wide range of environmental stimuli (Benedetti et al., 2008) suggest that optimal treatments should follow an individual optimization.

## III. Chronotherapeutics

Chronotherapeutics is the science of preventing or treating illnesses according to biologic rhythms (Halberg, 1973). It involves the timing of pharmacological and nonpharmacological interventions, such as surgery, physical agents, and psychotherapy. The goal is to minimize toxicity or adverse events, and/or to enhance treatment efficacy through adequate treatment timing and shaping (Sancar et al., 2015; Selfridge et al., 2016). More recently, chronotherapeutics has also aimed at targeting treatments toward the rhythm-generating biologic timing systems, to improve outcomes through amplification, dampening, alteration, or resetting. Although some treatment schedules involve the delivery of medications according to rhythms with periods usually ranging from 1 to 6 hours, so called ultradian (Belchetz et al., 1978; Chen et al., 2016), chronotherapeutics has mostly considered the implications of the CTS for treatment effects (Lévi and Schibler, 2007; Lévi et al., 2010; Lévi and Okyar, 2011; Dallmann et al., 2014, 2016). As a result, the current review focuses on circadian chronotherapeutics.

The demonstration that circadian rhythms were endogenous led to investigate the implications of the temporal organization of biologic functions for drug effects in preclinical models. Experiments in the 1960–1970s demonstrated up to several-fold reproducible changes in toxicity as a function of circadian timing of a fixed dose of methopyrapone, an adrenal cortical inhibitor (Ertel et al., 1964); morphine, an analgesic (Morris and Lutsch, 1967); lidocaine hydrochloride, an anesthetic and antiarrhythmic (Lutsch and Morris, 1967); ouabain, an antihypotensive (Nelson et al., 1971); methadone, an anti-addiction agent (Lenox and Frazier, 1972); arabinosylcytosine, an antimetabolite cytostatic (Haus et al., 1972); cyclophosphamide, an alkylating cytostatic (Haus et al., 1974); or lithium, an antidepressant and mood regulator (Hawkins et al., 1978). Although the potential implications for reducing adverse events of treatments were already emphasized, the results generated lively scientific controversies, which usually resulted from methodological issues regarding animal characteristics, synchronization, and manipulations (Lévi and Schibler, 2007). Their implementation has resulted in the demonstration of circadian time–dependent pharmacology for over 400 medications, including nearly 50 anticancer agents administered via i.v., i.p., s.c., or oral routes in mice or rats (Lévi et al., 2010; Dallmann et al., 2016).

### A. Chronopharmacology

The observed and measurable rhythms in drug tolerability and/or efficacy led to question the mechanisms at work regarding the relevance of both the rhythmic exposure to the drug and its metabolites (chronoPK) and the rhythmic organization of drug targets (chronoPD) (Reinberg and Halberg, 1971; Bruguerolle, 1998; Lemmer, 2005; Lévi and Schibler, 2007; Lévi et al., 2010; Lévi and Okyar, 2011; Dallmann et al., 2014, 2016).

#### 1. Chronopharmacokinetics

**Fig. 3.**
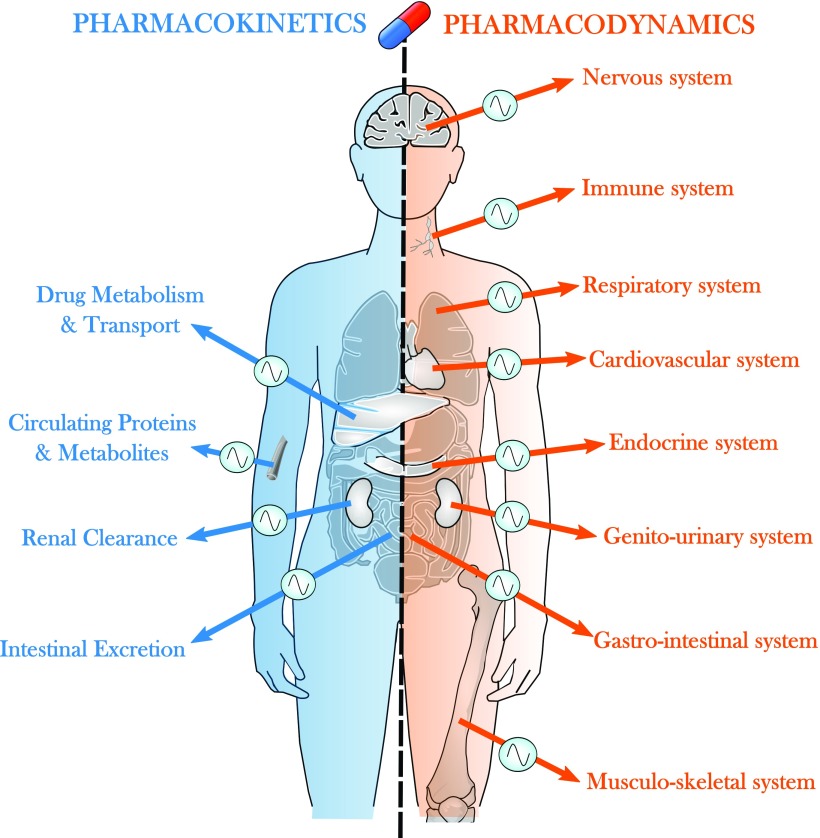
Circadian control of drug PK-PD. The CTS regulates drug transport at various levels, including intestinal absorption, intracellular uptake and efflux, and renal and intestinal excretion. Similarly, the amount of protein and metabolite binding to drugs in the plasma varies according to circadian time. Regarding PD, most systems of the organism are under the control of the CTS at the molecular, cellular, and physiologic levels. They can either be altered in specific diseases and impact on drug chronoefficacy or be involved in drug tolerability as targets of dose-limiting toxicities.

Twenty-four–hour changes have been demonstrated for each of the processes that determine drug disposition, that is, absorption, distribution, metabolism, and elimination and/or their toxicities (Fig. 3) (Bruguerolle, 1998; Lemmer, 2005; Lévi and Schibler, 2007). Such chronoPK moderate the exposure dynamics of target tissues to bioactive drug metabolites, irrespective of drug class, route of administration, residence time, or single versus repeated dosing schedule (Lévi and Schibler, 2007). However, the physicochemical properties of a medication can modify its absorption parameters and affect its dosing time dependency (Lemmer, 2005). Large circadian time dependencies can also characterize the pharmacokinetics (PK) of sustained release preparations of several medications both at steady state and during prolonged constant rate infusions. The 24-hour changes in absorption, distribution, metabolism, and elimination and/or their toxicities result from a host of physiologic rhythms, including gastric pH; gastric and small intestinal motility; plasma proteins and protein subtypes; membrane microviscosity; receptor density or binding enzymatic activities, transport proteins and ion channels, limb, liver, and renal blood flows; liver metabolism; and bile volume and salt excretion, as well as renal glomerular filtration rate, tubular reabsorption rate, and urinary output and pH (Lévi and Schibler, 2007; Dallmann et al., 2016). The relevance of circadian timing for drug persists in fasting rodents or humans. Yet, food intake or composition can modify the average PK parameters, yet only slightly alter the overall chronoPK profile (Bruguerolle and Prat, 1989). An imposed feeding pattern, however, can shift the synchronization of peripheral clocks, especially in the digestive system (Damiola et al., 2000), thus shifting the chronopharmacological profile of a drug accordingly.

#### 2. Cellular Mechanisms of ChronoPK

On the cellular level, the genetic clock directly controls the transcription of key rate-limiting steps in many metabolism pathways. Clock-controlled proline and acidic amino acid-rich basic leucine zipper transcription factors, such as albumin D-box albumin-binding protein, hepatic leukemia factor, and thyrotroph embryonic factor, further bind rhythmically to D-box–containing promoters of key genes that regulate xenobiotic metabolism. These include *PORs*, *ALAS*, *CAR*, *PPAR*, and *AhR*. Indeed, the expression of *CYPs*, *ALAS1*, and *POR* must be coordinated to permit efficient detoxification. Thus, the molecular clock redundantly and rhythmically controls Phase I reactions, including the *PORs* and the *CESs* (Gachon et al., 2006; Ballesta et al., 2011). The molecular clock further directly and indirectly controls the Phase II detoxification systems, including *UGTs*, N-acetyltransferases, and the reduced glutathione cycle (Gachon et al., 2006), as well as the Phase III transporters, such as *ABCB1* (P-gp), *ABCC2*, and *ABCG2* (Murakami et al., 2008; Zhang et al., 2009b; Okyar et al., 2011). Members of Phase I, II, and III families, as well as *ALAS1* and *POR*, displayed circadian expression at enzymatic activity, protein, and/or mRNA levels. A recent study showed that conditional deletion of *Bmal-1* in renal tubular cells of adult mice impacted both renal transcriptome and plasma metabolome and induced a decrease of 80% in the protein expression of organic anion transporter 3, which was paralleled by a reduced kidney excretion of the anionic drug furosemide (Nikolaeva et al., 2016). The circadian control of pharmacological enzymes may also originate from the masterclock as for CYP P450 genes in the mouse liver, which were found to be regulated through neuropeptide Y, the latter being driven by SCN-derived signals rather than by the cellular clock (Erion et al., 2016). Hence, the regulation of xenobiotic detoxification is complex, in that the expression of Phase I, II, and III components, *ALAS1*, and *POR* can be cell-type–specific, daytime-dependent, and substrate-inducible. Several of the above-mentioned transcription factors either accumulate in a circadian manner, display circadian activity, or are induced in a daytime-dependent manner (Richardson et al., 1998; Gachon et al., 2006).

#### 3. Chronopharmacodynamics

Drug activity is modulated by the circadian rhythms of 1) its direct intracellular target and triggered pathways and 2) the extracellular environment circadian status as a result of the control by the CTS of most physiologic functions, including the cardiovascular, immune and inflammatory, energy regulation, and nervous systems (Fig. 3). Studies involving the in vitro exposure of cells, obtained at different circadian times, to anticancer agents, have first identified cellular rhythms as major pharmacology determinants. As an example, bone marrow cells were sampled from mice at six different times. The cells were then cultured in the presence of different concentrations of theprubicin, a topoisomerase II inhibitor. Large-amplitude 24-hour rhythms characterized the in vitro cytotoxicity of this agent at several dose levels that were tested. The in vitro chronotoxicity of theprubicin for hematopoietic progenitors matched the in vivo hematologic chronotoxicity, following theprubicin i.v. delivery into mice (Lévi et al., 1988). Indeed, an endogenous circadian rhythm characterized the proliferative response of mouse bone marrow cells to granulo-monocytic colony-stimulating factor. Such chronopharmacology was demonstrated whether this hematopoietic growth factor was delivered to fresh bone marrow cells obtained at different circadian times, or to bone marrow cells cultured for up to 4 days and exposed at different circadian times (Perpoint et al., 1995; Bourin et al., 2002).

### B. Clinical Relevance of Chronotherapeutics

Several Phase III clinical trials testing chronotherapy versus conventional non–time-stipulated treatment schedules have resulted in up to fivefold better tolerability and a near doubling in efficacy (Dallmann et al., 2016). Meta-analyses of chronotherapy schedules have further suggested a survival benefit in male patients (Giacchetti et al., 2012). However, a number of randomized comparisons between morning and evening dosing times have shown similar rates of toxicities and/or efficacy for several drugs (Dallmann et al., 2016). These findings suggest that either the study design missed the optimal timing, excessive or insufficient dose levels were tested, or interpatient differences masked the circadian timing effects. For instance, some trials have assessed the effects of drug timing by grouping patients receiving drugs of the same class (e.g., angiotensin receptor blockers, calcium channel blockers), although each of the molecules in a given class likely presents different chronoPK-PD and chronotoxicity patterns as demonstrated for anticancer cytototoxic agents (Lévi et al., 2010; Stranges et al., 2015). Furthermore, experimental and clinical data have revealed broad interindividual CTS differences, resulting in different chronotoxicity profiles. Such differences can result from genetically determined chronotypes as well as from epigenetic changes, age, sex, lifestyle, disease, or pharmacological treatment, as discussed further in this review. Pioneering studies have, however, highlighted the relevance of morning dosing of glucocorticoids to minimize adverse events, resulting from adrenal suppression, resulting in the current timing recommendations for glucocorticoid intake in daily medical practice. Evening dosing has been recommended for most theophylline preparations to enhance bronchodilation and reduce side effects in asthmatic patients. However, morning dosing was shown to be more effective and safer for a sustained release preparation of theophylline (Smolensky et al., 2007). Similarly, evening dosing has been recommended for several anti- H1 and anti-H2 antihistamines in allergic and gastritis subjects, respectively, as being both more effective and better tolerated. For instance, the oral intake of 10 mg mequitazine provided more effective symptom relief than morning intake in a double-blind randomized trial involving patients with severe seasonal rhinitis (Smolensky et al., 2007). Clinical studies have also revealed the relevance of circadian rhythms for anticoagulant therapy in patients with thrombo-embolic disorders, while emphasizing the occurrence of rhythmic and nonrhythmic patients regarding heparin chronopharmacology (Decousus et al., 1985).

A recent study investigated the time of administration recommendations on chronotherapy for 30 commonly prescribed medicines in Australia (Kaur et al., 2016). In 56% of 27 research studies matching inclusion/exclusion criteria, the therapeutic effect of the medicine varied with the time of administration, that is, supported chronotherapy. For some medicines (e.g., simvastatin in the evening), circadian-based optimal administration time was evident in the information sources. Indeed, the circadian PK-pharmacodynamics (PD) of a drug can profoundly impact on its efficacy and tolerability, as illustrated for patients with cancer in Chapter 5, and rheumatologic, cardiovascular, or metabolic diseases in Chapter 6.

### C. Technology for Circadian Drug Delivery

Clinical chronotherapeutics have motivated both the development of programmable-in-time drug delivery pumps and the design of new drug formulations aiming at targeting specific circadian time windows. These recent technologies, together with the development of forecasting methods, are an important prerequisite for successfully translating the results of theoretical approaches into the clinics.

#### 1. Programmable-in-Time Infusion Pumps

Conventional infusion protocols of cancer chemotherapy only consider drug doses, duration, and frequency of infusions. As a result, treatment times often vary among and within patients, yet mostly between 9:00 and 17:00, that is, over only one-third of the day span, for hospital logistics reasons. In contrast, circadian chronomodulated schedules stipulate the time courses and parameters of the delivery profile for each anticancer medication over the 24-hour period to achieve the best therapeutic index, according to biologic rhythm-based specifications. This includes times of onset and offset of infusion and variation of flow rate, ranging from constant to sinusoidal or gradually increasing or decreasing. These new concepts of drug delivery have triggered the industrial development of nonimplantable multichannel programmable-in-time pumps, which in turn have fostered the clinical development of cancer chronotherapeutics. Multiple circadian infusion schedules are then jointly administered to nonhospitalized patients, with minimal or no medical or nursing intervention. The advent of the IntelliJect device with four 30-ml reservoirs enabled the development of the first combination schedule of 5-fluorouracil (5-FU)–leucovorin–oxaliplatin and led to the initial demonstration of the safety and efficacy of this three-drug chemotherapy given according to a circadian-chronomodulated delivery schedule, several years before the registration of oxaliplatin (Lévi et al., 2010). Melodie, a second generation of electronically engineered four-channel programmable pumps, represented considerable technological progress, through increased energy autonomy, flexible reservoir capacity, rapid programming of any delivery schedule, computer storage of treatment protocols and patient data, as well as actual drug delivery reports for each treatment course. The infusion pressure of this pump allowed the safe and effective administration of irinotecan–5-FU–oxaliplatin in a European trial involving conventional or chronomodulated three-drug infusions into the hepatic artery (OPTILIV) (Lévi et al., 2016). This device is currently being upgraded to become the first connected e-chronopump. Further applications are foreseen for chronic antibiotic or nutrition delivery, among others.

#### 2. Modified Release of Oral Drugs

Chronotherapeutics concepts have further elicited the development of cutting-edge technologies for modified release (MR) drug formulations aiming at selective tissue exposure at the desired time window over the 24 hours (Khan et al., 2009; Patel, 2015). For instance, the physiologic nocturnal high values of plasma melatonin were mimicked with Circadin, a melatonin formulation that releases this hormone over 5–7 hours following evening intake (Lemoine and Zisapel, 2012). Similarly, a MR formulation of prednisone was developed to achieve sustained low-dose tissue exposure during the early night span, following evening intake, and a rise in plasma levels starting near 4:00, to culminate around 8:00, and decreased gradually thereafter, thus mimicking the physiologic circadian pattern of cortisol secretion (Henness and Yang, 2013). Such chronomodulated release of prednisone would further counteract the proinflammatory cytokines that are usually released at night, and contribute to the early morning joint inflammation that characterizes rheumatoid arthritis. Indeed, MR prednisone decreased by 20% disease symptoms compared with placebo when associated to standard antirheumatic drugs and achieved a better reduction of morning stiffness compared with immediate-release prednisone (Henness and Yang, 2013). Other drug formulations aim at achieving a delayed peak exposure in the early morning when the drug is administered before going to bed to prevent acute events in the early morning. For instance, controlled pulsatile release capsules of montelukast sodium were developed for the prevention of episodic attack of asthma in the early morning and associated allergic rhinitis (Ranjan et al., 2014). It is also possible to combine several active compounds in the same formulation to insure specific delays in between each drug exposure. For instance, a multilayered multidisc tablet comprising two agents enveloped by drug-free barrier layers was developed in the context of chronotherapeutic disorders, employing two model drugs, theophylline and diltiazem, and provided two pulses of drug release (Khan et al., 2013). Apart from oral administration, transdermal technologies have been developed to achieve proper drug release timing according to skin temperature (Malik et al., 2008; Hammann et al., 2016). This formulation has the advantage to adapt to the individual patient’s temperature rhythms allowing personalized drug timing.

#### 3. Toward Rhythm-Sensing Drug-Releasing Nanoparticles.

Inter- and intrapatient variability critically impact on the tolerability and efficacy of drugs given at their recommended dose level. For instance, systemic drug exposure can vary more than 10-fold in individual patients, despite dose adjustment to body weight or surface area. Such variability greatly limits the success rate of pharmacotherapies. Although chronomodulated delivery at fixed time appeared to reduce such intersubject variability in maximum plasma drug levels, as shown for 5-FU and oxaliplatin (Metzger et al., 1994; Lévi et al., 2000), it did not eliminate CTS differences among subjects, resulting in important differences in drug elimination kinetics (Kwiatkowski et al., 2003). Novel nanotechnology-based approaches could link drug release to a relevant molecular circadian rhythm in the cells of interest. This would achieve effective delivery of chronotherapy according to individual patient rhythms independently from drug timing. Rhythmic trigger-elicited drug formulation could present a great benefit particularly in the field of cancer research, as anticancer chemotherapy commonly results in dose-limiting adverse events, thus favoring acquired resistance, poor efficacy, and poor patient outcomes.

## IV. Systems Approaches toward Personalized Chronotherapeutics

What is meant by systems approaches could be defined by the use of mathematical and statistical methods to analyze multitype and multiscale datasets. In the context of chronotherapeutics optimization, those pluridisciplinary pipelines mostly aim at designing patient-specific drug combinations and administration schedules. We first review the different mathematical models representing the circadian control of the following: 1) intracellular pathways within a single cell and 2) the dynamics of a whole cell population, in the absence of drug. Those models can represent either diseased or healthy cells/organs, and the next step toward therapeutic optimization consists in representing the drug chronoPK-PD on these tissues. We further explain why it is crucial to base the pharmacological modeling on the cell/organ physiology as it allows for multiscale approaches ultimately leading to reliable clinical models that provide the basis for a personalization algorithm.

### A. Multiscale Modeling of the Circadian Control of Healthy and Diseased Tissues

Mathematical modeling has aimed at knowledge improvement regarding the multilevel interactions between the CTS and the peripheral tissues to predict drug chronopharmacology and chronotoxicity. To ensure the clinical translation of experimental findings, multiscale methodologies are required in which living organisms are not subdivided down into independent components, but rather, it is recognized that genes, proteins, cells, and organs interact with each other and with the environment in complex ways that can vary over time (CaSyM, 2014). To properly address these issues, there is a need to consider first at the single-cell level the molecular circadian clock and the oscillatory dynamics generated in other cellular functions, such as the cell cycle, another critical determinant of many drug effects. Next, single-cell models need to be integrated into representations of cell populations to assess pharmacological effects at the tissue scale.

#### 1. Single-Cell Level

##### a. The molecular circadian clock

As described above, the molecular circadian clock is an intracellular network involving approximately 15 genes interlinked in several feedback loops resulting in an autonomous oscillatory system. Several mathematical models of the cellular clock have been developed involving different levels of complexity, reviewed in (Ukai and Ueda, 2010; Bordyugov et al., 2013; Ortiz-Tudela et al., 2013). Some models were based on delay differential equations (DDEs) when focusing on understanding the overall system dynamics with respect to the length of the delays in between molecular events, such as that from clock gene transcription to inhibition. However, those DDE-based models do not investigate the precise chemical reactions responsible for the delays so that models based on ordinary differential equations (ODEs) were developed that further represent the molecular events of clock gene transcription, translation, post-translational regulation, and degradation. Indeed, those equations represent the variations over time of intracellular mRNA or protein amounts explicitly computing the reaction rates of molecule interactions, production, transport, post-translational modifications, and/or degradation. ODE-based models and their comparison with experimental data in normal and knockout cell lines constitute a critical tool to investigate the structure of the clock, the involvement of particular genes, and the effect on the clock of specific gene mutations (De Maria et al., 2011).

The cellular clock generates in turn circadian rhythms in intracellular levels of many mRNAs and proteins by acting on either gene transcription, translation, post-translational regulation, or degradation. Although the molecular details of the clock control on those processes and their relative importance are known for certain genes (see *The Circadian Timing* *System and Its Multilevel Intersubject Variabilities*), more investigations are still needed for critical genes involved in drug PK-PD, and ODE-based models can in this study help generate experimentally testable hypotheses. For instance, topoisomerase 1 (TOP1) is an enzyme that relaxes supercoiled DNA and thus participates in important molecular processes along the cell cycle. TOP1 is also the target enzyme of the anticancer drug irinotecan, whose inhibition results in DNA breaks and cellular apoptosis. TOP1 mRNA and cytoplasmic protein levels displayed circadian rhythms in synchronized cell cultures of a human colorectal cancer model at confluence (Dulong et al., 2015). Although it has been shown that the protein dimer CLOCK-BMAL1 promotes *TOP1* transcription, the circadian regulation of *TOP1* expression remains still mostly unknown. The model of the molecular clock by Leloup and Goldbeter (2003) was supplemented to incorporate Top1 mRNA and protein dynamics, including the control by CLOCK-BMAL1. It was calibrated to experimental data on Bmal1, Per2, and Top1 mRNA levels and protein levels, and concluded that TOP1 protein degradation had to be under circadian control for the model to match the data (Hope and Ballesta, 2015). This hypothesis is plausible as TOP1 is degraded by the proteasome after ubiquitination, both processes being under the control of the circadian clock (Pommier, 2006). A full understanding of the molecular mechanisms involved in the control of Top1 expression would allow for a reliable prediction of the gene circadian rhythm according to the clock phase, hence of cell-type–specific irinotecan chronotoxicity.

##### b. The molecular clock and cell cycle as a coupled system

**Fig. 4.**
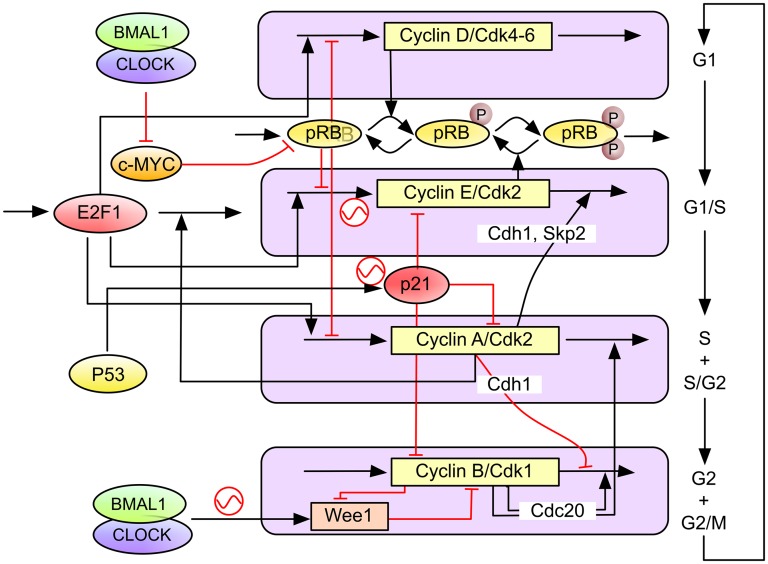
Molecular pathways of the circadian clock control on the cell cycle machinery (adapted from Gérard and Goldbeter, 2012). Several molecular processes along the cell cycle are regulated by the clock. At the early G1 phase, the BMAL1/CLOCK heterodimer downregulates Myc transcription to prevent its overexpression. In response to double-strand DNA damage, PER1 directly interacts with ataxia telangiectasia mutated and CHK2 to control G1 checkpoint. DNA damage induced by *γ*-radiation activates ataxia telangiectasia mutated/CHK2-mediated G1/S and G2/M checkpoints via p53 and p21. DNA damage induced by UV radiation leads to activation of ATR/CHK1-mediated intra-S checkpoint. In S phase, CRY2/TIM complex directly interacts with ATR/CHK1. In the G2 phase, PER-mediated ataxia telangiectasia mutated/CHK2/p53 signaling in response to DNA double-strand breaks leads to activation of G2/M checkpoint. BMAL1/CLOCK-activated Wee1 expression leads to activation of G2/M checkpoint.

A number of genes controlling the key steps of initiation, progression, and checkpoint functions of the cell cycle clock have been identified as being clock-controlled (Table 1). The circadian clock regulates the cell cycle by transcriptional control or direct protein–protein interactions (Fig. 4), including intracellular signaling (Matsu-Ura et al., 2016). For instance, in G1, the cyclin-dependent kinase inhibitor *P21* is transcriptionally regulated by clock genes *REV*-*ERBα* and *RORα/γ* (Grechez-Cassiau et al., 2008), whereas, at the G1/S transition, the transcription and RNA splicing encoding gene NONO regulates the p16-Ink4A checkpoint gene in a PER-dependent fashion (Kowalska et al., 2013). Transcription of the *WEE1* kinase (G2/M transition) is tightly controlled by the CLOCK:BMAL1 dimer (Matsuo et al., 2003). At the post-translational level, CRY modulates check (CHK)1/ataxia telangiectasia and Rad3 related (G1/S transition checkpoint) by interacting with Timeless in a time-of-day–dependent manner. PER and Timeless also regulate the G2/M transition via interactions with CHK2-ATM (Unsal-Kaçmaz et al., 2005; Kondratov and Antoch, 2007; Yang et al., 2010; Kang and Leem, 2014). Other clock-controlled cell cycle regulators include known oncogenes (c-MYC, MDM2, and *β*-catenin), cyclins (cyclin D1, B, and A), and TP53 (Gotoh et al., 2016; Huber et al., 2016). In particular, c-MYC, which plays a key role in G1 cell cycle initiation as well as cell growth and death, is directly transcriptionally regulated by BMAL1/CLOCK and BMAL1/NPAS2 via the two E-boxes in its P1 promoter. In contrast, deregulated expression of c-MYC disrupts the molecular clock in vitro by directly inducing REV-ERB*α* to dampen expression and oscillation of BMAL1 (Altman et al., 2015). Many key cell cycle regulators, such as cyclin-dependent kinase 4, integrin subunit α6, Wingless-type mouse mammary tumor virus integration site family, member 3, LIM Homeobox 2, transcription factor 4, Sex determining region Y box, SMAD7, and YB-1, are also directly clock-regulated (Fu and Kettner, 2013; Pagano et al., 2017).

**TABLE 1:** Cell cycle components regulated by the mammalian circadian clock

Clock Regulators	Cell Cycle Targets	Mechanism	Cell Cycle Event	Reference
CLOCK:BMAL1	WEE1	Transcription	G2/M	Matsuo et al., 2003
REV-ERB*α*	P21	Transcription	G1	Grechez-Cassiau et al., 2008
NONO	P16/INK4	PPI	G1	Kowalska et al., 2013
DEC1	cMYC	Transcription	G1	Sun and Taneja, 2000
PER1	ATM, CHECK2	PPI	DNA damage	Gery et al., 2006
PER2	TP53	PPI	DNA damage	Gotoh et al., 2014
CRY2/TIMELESS	ATR, CHECK1	PPI	DNA damage	Unsal-Kaçmaz et al., 2005
CSNK1D	WEE1	Phos	G/2M	Penas et al., 2014
CSNK1E	CDC25	Phos	G2/M	Piao et al., 2011

Phos, phosphorylation; PPI, protein–protein interaction.

It is reasonable to hypothesize that these regulatory links constitute the molecular basis for a tight coupling between the circadian clock and cell cycle networks that would enable these two oscillators to synchronize and thus coordinate the cellular processes that they control. However, it is very difficult to see how to decipher the dynamic functionality of these molecular interactions using classic biochemical and genetics approaches alone, and the need for modeling and single-cell imaging is obvious.

One-to-one phase locking of oscillators is a well-known dynamical phenomenon in which two coupled oscillators have a fixed relative phase and thus oscillate with a common frequency (Guckenheimer and Holmes, 1983). A necessary condition for two oscillators to lock in this way is for their natural frequencies, when uncoupled, to be close and for them to be coupled strongly enough. Therefore, it is reasonable to expect that functional links as above should lead to 1:1 phase locking of the clock and cell cycle when their uncoupled periods are similar. Indeed, in theoretical studies, such phase locking has been shown for mechanistically detailed mathematical and automaton models of the mammalian systems (Zamborszky et al., 2007; Altinok et al., 2011; Gérard and Goldbeter, 2012). This has recently been investigated by quantifying the dynamics of the two oscillators in real time, in single live mammalian cells (Bieler et al., 2014; Feillet et al., 2014). Both studies used the circadian clock reporter REV-ERB*α*::VENUS (Nagoshi et al., 2004). For cell cycle, Bieler et al. (2014) scored timing of division, whereas Feillet et al. (2014) added the fluorescent ubiquitination-based cell cycle indicator cell cycle reporter system (Sakaue-Sawano et al., 2008) probing cell cycle progression. These fluorescent markers were used to quantitatively determine the properties of each oscillator in single NIH3T3 mouse fibroblasts. Time lapse imaging combined with extensive statistical analysis and modeling exposed the dynamical properties of these two biologic oscillators.

The results depended upon whether the cells were synchronized using a 2-hour treatment with dexamethasone or were left unsynchronized. When neither clock nor cell cycle was synchronized by external cues, the cells appear robustly coupled with a 1:1 ratio between their respective periods over a wide range of observed periods (18–27 hours). A clear shortening of the circadian period occurred in dividing cells compared with nondividing cells, thus revealing an influence of cell cycle on the clock. Mathematical analysis and stochastic modeling unambiguously showed that phase locking rather than gating governs the interaction in NIH3T3 cells. Thus, the phases of the clock and cell cycle are coordinated all the way around the circadian cycle. Changing cell cycle duration impacted on circadian cycles, but 1:1 locking was resilient to such changes (Bieler et al., 2014; Feillet et al., 2014). Additionally, inhibition of the cell cycle at the G1/S or G2/M transitions lengthened circadian intervals and delayed division phase. Bieler et al. (2014) looked at the reverse interaction by changing circadian period. This did not affect cell cycle length, but advanced division with respect to circadian phase. The authors thus proposed a unidirectional coupling from the cell cycle to the circadian clock (Bieler et al., 2014), but this experimental result is also compatible with bidirectional coupling.

When, in contrast, the cells had a 2-hour treatment with dexamethasone, which resets the circadian clock, two distinct dynamical behaviors were observed (Feillet et al., 2014). Whereas one subpopulation kept a 1:1 phase locking, outside this the ratio of cell cycle and clock periods was different and often in a ratio p:q (i.e., p cell cycles for q clock cycles), where p and q are small integers. For example, when the cells were grown in rich 20% fetal bovine serum (FBS) culture medium, p:q was 3:2, and, when this was reduced to 10% FBS, p:q = 5:4 was observed. This is compatible with the way that increased FBS was observed to speed up the oscillations. Moreover, when projecting the timing of mitosis across the whole experiment, a clear clustering of cell division was observed, suggesting that the cell cycle was synchronized by physiologic cues via the circadian clock, again supporting bidirectional coupling. This behavior is entirely in accordance with what would be predicted from the mathematical theory for coupled deterministic oscillators.

Introduced in Mori et al. (1996), gating is defined as clock-based control of cell division which is allowed at certain clock phases and forbidden during others, thus creating proliferation checkpoints. The gating model differs from the phase-locking model in which, in noise-free systems (and approximately in stochastic systems), the two oscillators are synchronized over the whole period so that observing the phase of one system provides information on the phase of the other. The above studies suggest the rejection of the concept of gating of the cell cycle by the clock in mammalian cells in favor of phase locking and indeed in the movies of Feillet et al. (2014), showing how the cell cycle and clock phases progress in single cells; there is no evidence of cells queuing to get through a gate. In the end, it seems that the cell cycle is capable of impacting on the circadian clock and vice versa, the dominant influence being dependent on the environment of the cell. Phase locking is a characteristic phenomenon of coupled oscillators and is likely to be a much-used mechanism used to function coordinate different cellular oscillators.

A major impact of clock and cell cycle coupling on cell physiology resides in timed mitoses (e.g., about one-sixth of human epidermal cells divide daily) in that local intracellular clock/cell cycle coupling most likely governs rhythmic mitosis at the cellular and tissue levels, whereas systemic circadian cues are required to coordinate cell divisions in the whole organism. An important example of clock control of the cell cycle that addresses this hypothesis from a different angle is provided by the discovery that different populations of epidermal stem cells express clock genes in opposite phases. This results in a differential propensity for activation, and it has been suggested that this heterogeneity may have evolved to allow the cells both to self-renew, thus replenishing their reserve in the niche, and to keep a ready-to-go population that can respond to the signals that trigger differentiation. Specific disruption of the circadian clock in these cells led to premature epidermal ageing, which confirms that local coupling is necessary to ensure tissue integrity (Janich et al., 2011). The interplay between the clock and cell cycle is of primary relevance to cancer because disordered circadian function has been implicated in the pathogenesis of cancer, and a deregulated cell cycle is a hallmark of cancer cells (Lévi and Schibler, 2007; Hanahan and Weinberg, 2011). Among the hallmarks of cancer, genome instability and mutations in cell cycle genes are a recurring enabling factor (Hanahan and Weinberg, 2011), with mutations in cyclin, cyclin-dependent kinase, or cyclin-dependent kinase inhibitor genes found in 90% of human cancers (Bonelli et al., 2014). In contrast, evidence is increasing that cancer cells also display a deregulation of the circadian clockwork, which may promote abnormal proliferation (Fu and Kettner, 2013).

#### 2. Cell Population Dynamics

The final objective of systems chronopharmacology lays in the prediction of the drug effect on a whole tissue rather than at the level of a single cell. Hence, several mathematical approaches were undertaken to model cell proliferation and its circadian control at a cell population scale, and efforts have been made to link those models to the single-cell representations as in a multiscale pipeline.

##### a. Age-structured partial differential equation and DDE models

Models of the dynamics of a cell population were designed based on partial differential equations (PDEs) incorporating both time and the age of the cells in their current cell cycle phase as structure variables (Billy et al., 2013, 2014; El Cheikh et al., 2014). Conversely to classic ODE-based cell population models, those equations present the advantage of imposing a minimum duration for cell cycle phases, which is an important physiologic feature to predict cell dynamics. Intercell variability in cell cycle phase durations followed a γ distribution in unsynchronized NIH3T3 in vitro experiments, which can be implemented in those models by choosing the corresponding phase transition functions (Billy et al., 2014). Those PDE models can also take into account the circadian control of both cell death pathways—though oscillating death rates—and of cell cycle phase transition and checkpoints—through transition functions displaying 24-hour rhythms. Starting from these PDE-based models and assuming no intercell variations in phase durations, DDEs can also be derived to model circadian-controlled cell proliferation (Bernard et al., 2010). In this case, the delays correspond to the common length of the cell cycle phases.

The main advantage of those types of models resides in the small number of parameters to estimate from data and the possibility to represent long-term behaviors and further derive theoretical conclusions. Recently, El Cheikh et al. (2014) also combined an ODE-based model representing the molecular interconnections between the circadian clock and the cell cycle to a PDE-based cell population model. They showed that the clock/cell cycle coupling increases the growth rate of cell populations for autonomous cell cycle length around 24 hours and above 48 hours. Moreover, they predicted that CRY1/CRY2 mutations decreased the cell population growth rate for all periods of the cell cycle, which was in agreement with lower liver regeneration potency experimentally found in CRY mutant mice. The loss of functional PER2 was predicted to lead to an enhanced proliferation, which is consistent with PER2 being reported as a tumor suppressor gene. Finally, BMAL1 knockout also increased the growth rate for cell cycle length smaller than 21 hours and decreased it elsewhere.

##### b. Agent-based models

An alternative approach to predict cell population dynamics consists in representing each cell individually although so-called agent-based models or cellular automaton. A combination of logical rules and intracellular ODE model simulations takes as input cues the cell spatial and chemical environment to ultimately define its behavior. The cell population dynamics is thus computed by assessing those rules for each considered cell, and intercell stochastic variability is often assumed in particular in gene expression. This type of modeling presents the advantage of being very flexible and can thus faithfully represent the biology. However, its computational cost can be very high because it proportionally increases with the number of cells considered. As an example, Nguyen et al. (2013) developed such a model to represent the circadian dynamics of inflammatory response after endotoxin administration in vivo, taking into account the cell-to-cell communication and intercell stochasticity. In silico experiments suggested that cell-to-cell synchronization in the leukocyte population was enhanced after endotoxin exposure.

### B. Multiscale Physiologically-Based ChronoPK-PD Modeling toward Therapies Personalization

Modeling of healthy and diseased tissues in the absence of drugs, as presented above, needs to be supplemented with PK-PD models to allow for pharmacotherapy optimization. Although classic compartment-based PK-PD modeling has a strong descriptive value in particular regarding interpatient differences, their predictive power is weak so that new mathematical methods are needed. Physiologically-based models representing the molecular mechanisms involved in drug chronoPK-PD have gradually appeared as critical tools to predict drug efficacy and side effects.

#### 1. What Is Physiologically-Based PK-PD Modeling?

Drug toxicity and efficacy are ultimately determined by the gene and protein networks involved in cellular, organ, and whole-body PK-PD. Hence, modeling the dynamics of the key molecular events constitutes a rational basis for treatment optimization. Thus, physiologically-based pharmacology modeling, first introduced by Rowland (1984), has rapidly developed over the past years, resulting in the implementation by pharmaceutical industries of dedicated clinically-focused software such as PKSim (Bayer) and Simcyp (Certara). Physiologically-based modeling consists in the quantitative representation of the molecular pathways involved in drug pharmacology and efficacy. These models are based on ODEs and represent in each considered physical compartment the following: 1) the concentrations of the parent drug and its metabolites over time, resulting from biochemical events such as bioactivation, detoxification, passive diffusion, or active transport; 2) the drug activity on the cells, such as pathway activation, DNA damage leading to DNA repair, cell cycle arrest, and/or cell death. Hence, all model variables and parameters do have a physical or biochemical significance, which allows for direct comparison with corresponding experimental or clinical measurements.

#### 2. Multiscale Approach To Design Physiologically-Based Patient Model

**Fig. 5.**
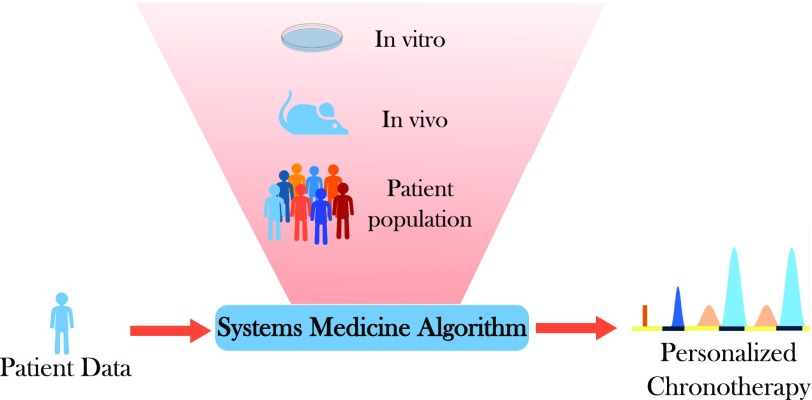
A systems medicine approach for personalized chronotherapeutics. Individual patient data—such as measurements of circadian biomarkers, gene polymorphism, patient general characteristics, or disease history—are input into the systems medicine algorithm that computes personalized chrono-infusion schemes. The algorithm is developed through a multiscale pipeline integrating mathematical and experimental investigations in cell culture, in laboratory animals, and in patient populations. Results in multiple cell lines, animal strains, and patient subgroups allow for the reliable design of the personalization framework.

The outcome of pharmacotherapies has long been known to depend upon both patient- and disease-specific genetic, epigenetic, or behavioral specificities. Thus, treatment personalization is required to ensure optimal health management. The fact that physiologically-based models integrate the molecular details of drug PK-PD allows one to explicitly integrate patient- or disease-specific molecular and lifestyle information. However, the temporal and spatial organizations of such complex physiology cannot be exhaustively measured in individual patients, due to the invasive and sometimes ethically questionable nature of the required clinical sampling procedures. Indeed, physiologic models involve a large number of kinetic parameters, which is often considered as the main drawback of the approach. Such limitations can be palliated by the use of a multiscale systems medicine approach, which integrates experimental results obtained in cell cultures, laboratory animals, healthy human subjects, and patient populations (Fig. 5). Indeed, because the models are based on the physiology, submodel structure and parameter values are conserved and can be either directly inferred or scaled on physiologic basis from one scale to another. Such pipeline thus allows for the design of a patient-specific PK-PD model that provides the basis for chronotherapeutics personalization.

Multiscale chronoPK-PD approaches start with in vitro investigations either in solutions or in cell culture to design and calibrate a detailed molecular chronoPK-PD model at the cellular level. Then mouse studies serve as a basis to design the drug-specific structure of the whole-body PK-PD model, which incorporates the cellular model to represent each considered organ. Next, a model for an average patient can be obtained by keeping the structure of the mouse model and resizing the parameters for humans using physiologic literature information combined to clinical datasets in patient populations. More precisely, model parameters can be scaled from mouse to human as follows: 1) organ volumes are inferred from literature values for each species according to age and sex (Davies and Morris, 1993; Marino, 2012); 2) intracellular reaction rates are kept unchanged from the preclinical models; 3) protein activities are proportionally scaled according to interspecies in vitro studies (Davies and Morris, 1993; Satoh and Hosokawa, 1998; Maier-Salamon et al., 2011); 4) blood-to-organ transport and drug clearance parameters can be scaled using physiologic information such as volumes or blood flows, although more work is needed in this area to develop a validated scaling method. Sensitivity analyses are then performed to determine the relative importance of model parameters and select the most influential; ones that will be modified for obtaining a patient-specific model. Hence, this analysis may also guide the search of relevant circadian or pharmacological biomarkers to be measured in patients. The human model is then partially recalibrated using individual patient datasets and further serves in optimization procedures to compute personalized administration schemes.

As an example, we detail in this work the design of a physiologically-based model of temozolomide (TMZ) brain disposition through a multiscale pipeline integrating PK studies in buffer solutions, cell culture, mice, and patients (Ballesta et al., 2014). As TMZ PK is pH-dependent, it was first studied in buffer solutions. TMZ conversion into 5-(3-methyltriazen-1-yl)imidazole-4-carboxamide (MTIC) and MTIC subsequent fragmentation into 4-amino-5-imidazole-carboxamide (AIC) and a methyldiazonium cation—the highly-reactive active species responsible for DNA adducts—were represented in a first mathematical model (three variables, four parameters), which was calibrated to experimental results on TMZ, MTIC, and AIC levels in solutions at different pHs. Next, TMZ cellular PK was represented through two compartments representing the extra- and intracellular medium, both incorporating the model of the buffer solution studies. Thus, the only parameters left to estimate were those associated to drug cellular uptake and efflux. They were estimated from TMZ, MTIC, and AIC concentrations measured in U87 glioma cells and corresponding extracellular medium. Regarding TMZ PD, DNA adducts formation by the active cation was represented through a linear kinetics involving one parameter estimated from literature data. To account for interlaboratory differences and to allow for a better fit of the cell culture data, a 50% deviation was allowed for the four parameters of the solution study whose data were obtained from a different research group. Next, a model of TMZ brain disposition in mice was developed incorporating the cellular model. The extra- and intracellular compartments of the latter now correspond to the interstitial fluid and tumor cells within the brain tumor. The normal brain was represented in the same manner and served as a control for the cancer compartments. A compartment for the blood was also added to account for systemic drug administration, and TMZ blood PK was modeled by a forcing function independently fitted to TMZ plasma PK in normal nude mice. Tumor cell membrane transport parameters were scaled from the in vitro investigation using volumes, whereas all intracellular parameters were kept unchanged. The six remaining parameters for 1) normal brain cell membrane transport and 2) transport between the blood compartment and the interstitial fluids were estimated from PK data in normal and U87 tumor-bearing nude mice. Finally, the human model was obtained by keeping the mouse model structure and intracellular parameter estimates and scaling all transport parameters proportionally to volumes. However, a comparison of this naive model to TMZ concentration measurements in the interstitial fluid of cancer patients revealed that the model overestimated drug concentrations by up to fivefold, thus advocating for a refined scaling method. Moreover, this model of TMZ PK can be extended to account for the circadian rhythms of drug transport and plasma protein binding.

#### 3. Optimization Procedures toward the Design of Personalized Pharmacotherapies

For a given drug, a patient-specific physiologically-based chronoPK-PD model is a critical tool, as it can theoretically predict the efficacy and toxicities of any administration schedules in the individual patient. Once it is designed and validated, the next step consists in utilizing it in optimization procedures to compute patient-tailored chronotherapies. The chronotherapeutics optimization problem can be formulated as an objective function (e.g., maximizing efficacy on cancer cells) subject to constraints (e.g., tolerability thresholds). Then optimization methods can be implemented to find the parameters of the single-agent administration timing (dose, duration, circadian time, ...) that achieve the optimal value of the objective function (Basdevant et al., 2005; Ballesta and Clairambault, 2014). This methodology can also serve to optimize the combination of the single agent with molecules targeted to proteins represented in the chronoPK-PD model (Ballesta et al., 2013). The targeted agent is represented in the model as a modification of the corresponding intracellular or systemic protein levels. Those parameters are then included in the optimization procedures that compute the optimal administration scheme of the single agent together with the optimal protein levels. This method only represents the PD of targeted molecules and aim at drug selection. Complete optimization requires representing the cellular and whole-body chronoPK of the targeted molecules to predict proper timing.

Optimal control theory and a descent algorithm have been applied to improve oxaliplatin chronomodulated delivery schedule along these lines (Basdevant et al., 2005). In this study, a simplified model of oxaliplatin PK-PD was developed based on both drug jejunal chronotoxicity and antitumor chronoefficacy. When aiming at eradicating the tumor under a constraint of tolerability, the theoretically optimal drug administration was a nontrivial chronomodulated drug infusion flow whose shape was critically determined by the numerical value of the toxicity threshold. Moreover, constant rate infusions always achieve worse therapeutic outcomes than optimized time-scheduled regimens in these models.

An alternative methodology consists in solving the optimization problem through numerical algorithms. An in vitro proof of concept of this approach was recently provided in NIH3T3 mouse fibroblasts either normal or modified to overexpress the oncogene SRC, the latter being considered as the cancer cells and the former as the healthy cells. In this experimental setting, the exposure of both cell populations to the same doses of various anticancer drugs combined or not to SRC-targeted molecules resulted in a moderate efficacy in transformed cells and an unacceptable cytotoxicity in normal fibroblasts. Physiologically-based modeling informed by protein level quantification in both cell types was used in optimization procedures, which allowed for the identification of nonintuitive anticancer drug combinations and scheduling inducing apoptosis in cells mutated for the oncogene SRC, but not in normal cells. The optimal combination chemotherapies relied on the administration of a cytotoxic drug and a SRC-targeted molecule combined to an inhibitor of the proapoptotic protein BAX, which was surprisingly more expressed in transformed cells compared with normal ones. Decreasing BAX level by the same quantity in both cell populations allowed for sheltering of healthy cells that could not trigger apoptosis anymore in the absence of BAX, whereas cancer cells were still drug sensitive because their initial BAX level was higher. The same optimization methods were further used for the optimization of irinotecan chrono-exposure in cell culture, described in this work after (Ballesta et al., 2011).

## V. Cancer as a Driver for Systems Chronotherapeutics

Proper timing of chemotherapies is particularly relevant for anticancer drugs, which are often administered near their maximum-tolerated dose, thus inducing adverse events, whose severity could be minimized by targeting specific times of day. Similarly, radiation therapy’s therapeutic index could be improved by optimizing timing of administration (Chan et al., 2016, 2017a,b). Moreover, tumor tissues usually display a disrupted circadian organization—at least at an advanced stage (Kettner et al., 2014). The difference in circadian synchronization between healthy and cancer tissues can then be exploited in treatment timing to specifically shield healthy cells while targeting cancer cells.

### A. Preclinical and Clinical Cancer Chronotherapeutics

#### 1. Preclinical Drug Chronotolerance and Chronoefficacy

Preclinical investigations performed in mice or in rats have demonstrated that circadian timing largely modifies the toxicity of nearly 50 anticancer drugs, including cytostatics, cytokines, and targeted molecules (Lévi et al., 2010). Rodent survival and body weight loss varied from 2- to 10-fold, according to circadian timing of the same drug dose. The circadian times of least toxicity were staggered along the 24-hour span and differed for molecules in the same pharmacological class advocating for systems molecular chronopharmacology to predict optimal timing (Lévi et al., 2010). The chronotoxicity rhythms arose from the circadian control of PK-PD and cytotoxicity. First, timing-dependent blood and/or tissue PK were observed in rodents for 17 anticancer drugs. Such chronoPK rhythms not always coincided with chronotoxicity patterns, thus attesting to the existence of circadian rhythms in cellular drug target, DNA repair, cell cycle, and/or cell death pathways (Lévi et al., 2010; Sancar et al., 2015).

Next, 28 anticancer molecules presented time dependencies in their antitumor efficacy of various amplitudes as measured by survival of tumor-bearing mice and tumor growth rate (Lévi et al., 2010). The chronoefficacy rhythms may at least partly originate from the circadian control of drug metabolism and distribution at the whole-body level, which influences tumor drug concentrations. In contrast, in vitro studies have shown that some cancer cell lines, such as human breast cancer MCF-7, have lost circadian rhythmicity in clock gene transcription (Xiang et al., 2012), whereas others such as human colon cancer Caco-2 at confluence have retained coordinated circadian expression (Dulong et al., 2015). However, most studies in vivo have shown the lack of consistent 24-hour patterns in experimental cancer tissues, especially for poorly differentiated carcinomas (Lévi et al., 2010).

Recent mouse investigations have highlighted large sex- and strain-related differences in drug chronopharmacology and chronotoxicities. The mRNA circadian expressions of genes involved in the anticancer drug irinotecan chronopharmacology, including *CES2* (bioactivation), *UGT1A1* (detoxification), *ABCC2* (transport), and *TOP1* (target enzyme), as well as irinotecan pharmacokinetics, differed according to sex and strain (Ahowesso et al., 2010; Okyar et al., 2011). Next, a large prospective investigation involved eight mouse categories, including two with clock gene *PER2* mutation, and revealed an 8-hour difference in the optimal timing of irinotecan. Twenty-seven circadian time series of mRNA gene expressions in the liver and in the colon mucosa were analyzed, although sparse linear discriminant analysis and the circadian transcriptional patterns of *REV-ERBα* and *BMAL1* best discriminated between the chronotoxicity classes. Further analysis through maximum a posteriori Bayesian inference allowed the design of a linear model of *REV-ERBα* and *BMAL1* circadian mRNA expressions, which accurately predicted the irinotecan chronotoxicity pattern (Li et al., 2013). Overall, these results emphasized the importance of mouse sex and strain in chronotherapeutics studies, together with the relevance of tracking circadian clock biomarkers for predicting optimal timing in different individuals, despite exposure to the same environmental synchronizer.

#### 2. Current Challenges of Cancer Chronotherapeutics in the Clinics

Clinical investigations, including randomized Phase III trials, have tested the timing effects of several anticancer chemotherapies. Proper circadian timing improved treatment outcome achieving an up-to-fivefold decrease in drug toxicity and nearly twofold increase in antitumor efficacy compared with noncircadian-based administration of the same drug doses (Lévi et al., 2010; Dallmann et al., 2016). However, recent clinical studies have demonstrated that the patient’s sex and genetic background have a large influence on the optimal timing of drug administration (Giacchetti et al., 2012; Roche et al., 2014). Nowadays, a unique chronotherapy infusion scheme directly inferred from mouse studies is administered to all patients, which results in a large interpatient variability in treatment outcome. In the case of metastatic colorectal cancer (mCRC), the fixed three-drug chronotherapy schedule ChronoFLO4 increased the overall survival of male patients compared with constant or other conventional administration method of the same drug doses, but decreased that of female patients according to a meta-analysis of three randomized international trials (Giacchetti et al., 2012). Interestingly, metastatic adenocarcinoma patients who did not tolerate an administration of 5-FU at 3:00 to 4:00 AM benefited from an infusion of the same drug at 9:00 to 10:00 PM (Bjarnason et al., 1993). Overall, these results suggest that personalized chronotherapy, designed through dedicated systems medicine approaches, is likely to increase patient’s response to treatment.

**Fig. 6.**
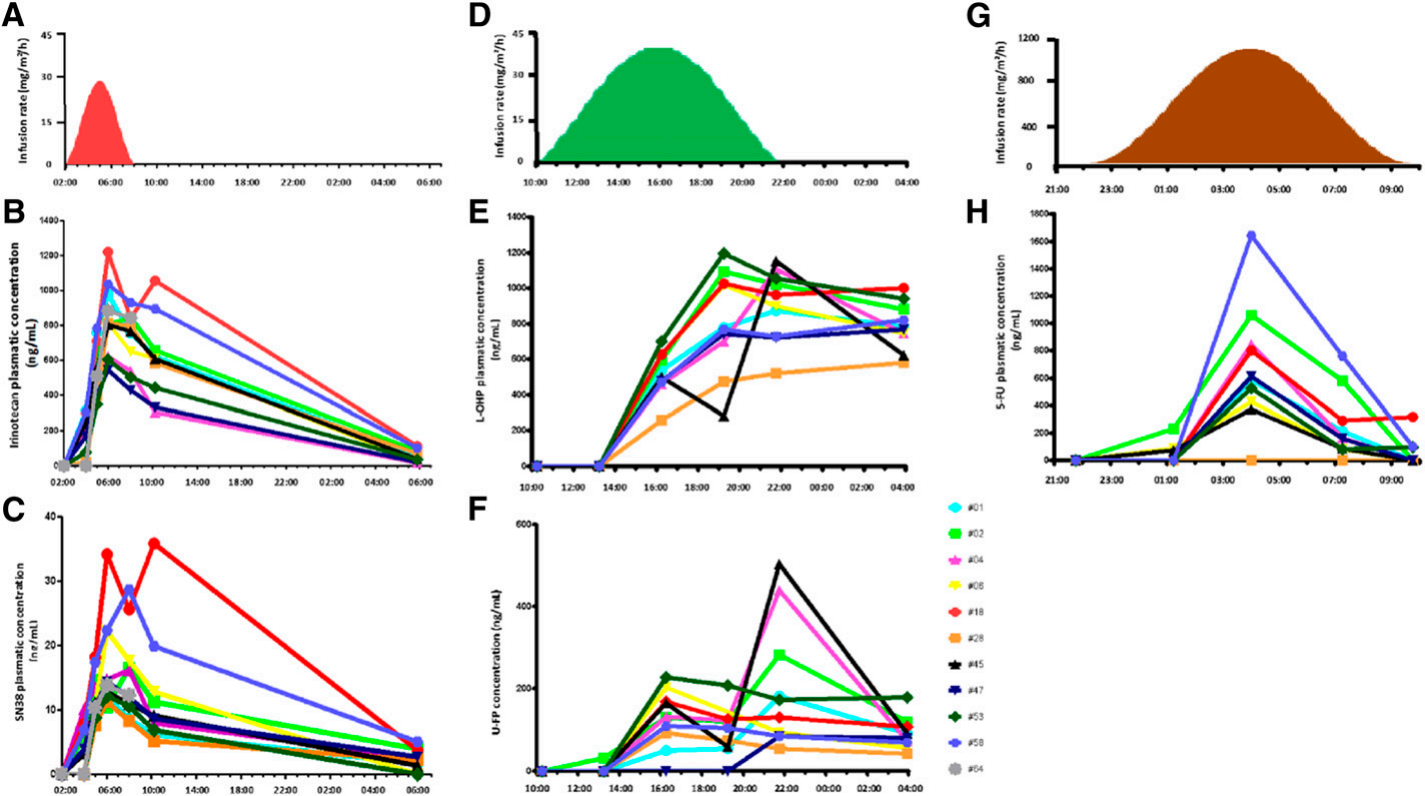
Relationship between hepatic chronomodulated delivery schedules and plasma PK profiles in metastatic cancer patients from the Optiliv trial. Irinotecan (A–C), Oxaliplatin (D–F), and 5-FU (G–H) were administered according to specified infusion patterns in the hepatic artery of 11 cancer patients. For irinotecan and oxaliplatin, a significant delay was observed between the administration peak time and the plasma PK curves of both administered agents and corresponding active metabolite or ultrafiltrate concentration. On the opposite, 5-FU plasma concentration closely followed the infusion profile for all patients. Large interpatient variability was observed in the plasma PK of all measured quantities [data from Lévi et al. (2017)].

Although most chemotherapies are administered directly into the central blood circulation, hepatic administration of antitumor drugs to colorectal cancer (CRC) patients with liver metastasis have now appeared as a very promising strategy. The European phase II clinical trial OPTILIV successfully tested in CRC patients the hepatic artery infusion of the anticancer drugs 5-FU, oxaliplatin, and irinotecan, combined to the targeted molecule cetuximab (Lévi et al., 2016). Although patient’s survival of this trial exceeded by far the usual survival in similar patient populations, plasma PK measurements revealed that the chronomodulated administration schemes used for oxaliplatin and irinotecan resulted in systemic exposure outside the targeted therapeutic time windows in several patients (Fig. 6) (Lévi et al., 2017). Hence, systems approaches are required to optimize hepatic administration schedules. Moreover, large interpatient differences were demonstrated in the plasma PK profile of all four drugs, which correlated with toxicity severity for oxaliplatin, thus advocating for personalized hepatic administration schedules to be designed and tested.

### B. Anticancer Systems Chronopharmacology

Personalizing cancer chronotherapeutics requires an extensive molecular knowledge of anticancer drug chronopharmacology and chronotoxicity both in healthy and tumor tissues. Indeed, the understanding of molecular mechanisms governing drug PK-PD, cell cycle, cell death, and their circadian control allows for accurate predictions of drug chronotoxicity according to tumor mutations and patient-specific gene polymorphisms and chronotypes. Such complex phenomena may highly benefit from mathematical modeling and systems pharmacology approaches that guide experimental design, and further allow for predictions from preclinical studies of most influential genes as potential circadian biomarkers for the clinics. We in this work review systems chronopharmacology studies of irinotecan, 5-FU, and oxaliplatin, the three cytotoxic drugs constituting the gold standard treatment of colorectal cancer (CRC), the third most common malignancy worldwide (Siegel et al., 2016).

#### 1. Irinotecan

**Fig. 7.**
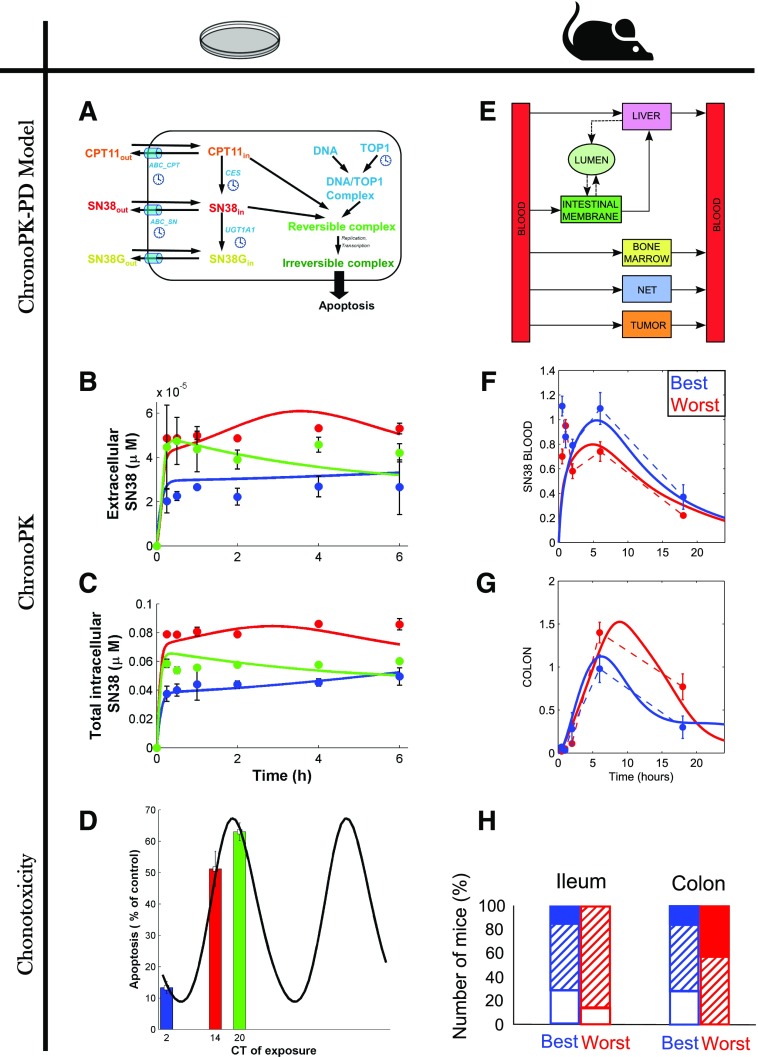
Multiscale systems chronopharmacology to personalize irinotecan chronotherapy. An in vitro study of irinotecan chronopharmacology led to the design of a cellular chronoPK-PD model (A) incorporating multitype experimental data, including the extra- and intracellular concentrations of active metabolite SN 38 and irinotecan-induced apoptosis after irinotecan exposure at three CTs (B–D) Dulong et al., 2015). This cellular investigation provided the basis for a mouse study and the development of a whole-body model of irinotecan chronoPK-PD explicitly incorporating the cellular model in relevant organs (E) (Ballesta et al., 2012). The model was first developed for B6D2F1 male mice in which several chronopharmacology datasets were available, including plasma and colon chronoPK profiles of SN38 after irinotecan at best and worst time of tolerability (F–G). The next step will consist in fitting intestinal chronotoxicity data available for the same mouse category (H) (Li et al., 2013). Dots or bars represent experimental results, and solid lines represent best-fit models.

Irinotecan is a topoisomerase inhibitor active against colorectal and gastrointestinal malignancies, yet with dose-limiting intestinal and hematologic toxicities. Irinotecan PK-PD, toxicities, and efficacy displayed circadian rhythms in synchronized cell culture, in mice and in cancer patients (Ballesta et al., 2012; Dulong et al., 2015). Irinotecan is usually administered to cancer patients mostly in combination with other cytotoxic drugs and/or targeted molecules (Lévi et al., 2010; Sasine et al., 2010). However, there is a critical need for a rational design of irinotecan-based chronomodulated drug combinations according to individual patient data (Campbell et al., 2017). To this end, in vitro and rodent systems chronopharmacology studies investigated the molecular determinants of irinotecan chronotoxicity and chronoefficacy (Fig. 7).

##### a. Irinotecan systems chronopharmacology in cell culture

Recent in vitro investigations have characterized irinotecan chronopharmacology at the molecular scale in human colorectal adenocarcinoma Caco-2 cells (Ballesta et al., 2011; Dulong et al., 2015). Experimental design was guided by an ODE-based physiologic PK-PD model that considered the following: 1) irinotecan passive diffusion through the cell membrane and bioactivation into SN38 through carboxylesterase; 2) SN38 detoxification into SN38G through UGT1As; 3) irinotecan, SN38, and SN38G efflux outside of the cells by ABC transporters. Regarding the PD part of the model, SN38 was assumed to stabilize its target TOP1 on the DNA, creating reversible complexes that become irreversible after collision with replication mechanisms. The amount of irreversible complexes was used as an output of irinotecan toxicity, as it was highly correlated with cell death in cell culture (Ballesta et al., 2011). Proteins involved in irinotecan efflux, bioactivation, detoxification, and the drug target TOP1 were assumed to display circadian rhythms.

**Fig. 8.**
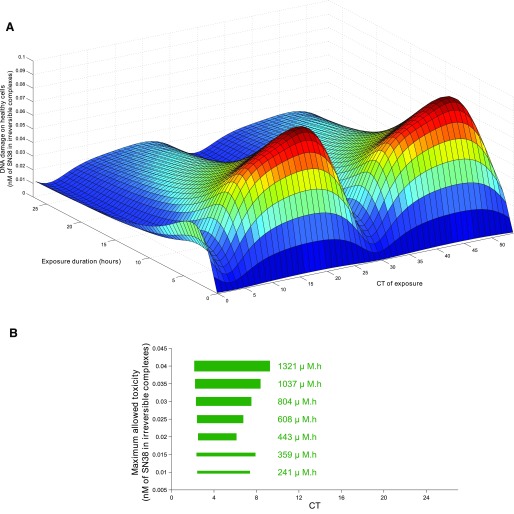
Optimizing irinotecan chronotherapy in Caco-2 cell culture (adapted from Ballesta **et** al., 2011). (A) Predicted drug cytotoxicity in synchronized cells with respect to exposure duration and circadian time of beginning of exposure. The cumulative dose was set to 500 mM/h. (B) Optimal exposure schemes following the strategy of maximizing efficacy in unsynchronized cells considered as cancer cells, under a constraint of maximal allowed toxicity in synchronized cells, considered as healthy cells. The toxicity threshold was varied (*y*-axis), and corresponding optimal schemes consisted in administering the optimal cumulative dose (written in green) over 3h40 to 7h10, starting between CT2 and CT3.

In the first study (Ballesta et al., 2011), circadian variations with a period of 26h50 (S.D. 63 minutes) were found in synchronized Caco-2 cells for the mRNA levels of the three clock genes *REV-ERBα*, *PER2*, *BMAL1*, and in that of *TOP1*, *CES2*, *UGT1A1*, and efflux transporters *ABCB1*, *ABCC1*, *ABCC2*, and *ABCG2*. DNA-bound TOP1 protein amount in the presence of irinotecan also displayed circadian rhythms. The model parameters were estimated from data, and the best-fit model closely reproduced the experimental datasets. Next, the data-calibrated model was used in numerical optimization procedures to compute theoretically-optimal exposure schemes for Caco-2 cells (Fig. 8). Considered schemes consisted of an in vitro exposure to a given concentration of irinotecan, over 1–27 hours, starting at a particular CT. Synchronized cells were considered as healthy ones, and nonsynchronized cells as cancer ones (Lévi et al., 2010). The optimization process aimed at maximizing drug efficacy on cancer cells under the constraint that toxicity in the healthy cell population remained under a fixed tolerability threshold. For all considered thresholds, the optimal exposure scheme consisted in administering irinotecan over 3h 40min to 7h 10min starting between CT 2h 10min and CT 2h 30min which corresponded to 1h 30min to 1h 50min before the nadir of carboxylesterase activation enzymes. The optimal schemes were not centered on the nadir of the latter rhythm but rather extended after it, when efflux transporters and deactivation enzymes were higher and therefore protected more efficiently healthy cells. The optimal schemes induced twice as much DNA damage in cancer cells as in healthy ones. Of note, the optimal duration did not exceed, highlighting the need for short exposure durations to optimally exploit the temporal difference between healthy and cancer cells.

Irinotecan chronopharmacology was further investigated in a second study in synchronized Caco-2 cells (Dulong et al., 2015). Consistently with the first study, large transcription rhythms of period 28 hour 06 minute (S.D. 1 hour 41 minute) moderated irinotecan bioactivation, detoxification, transport, and target. These molecular rhythms translated into statistically significant changes according to drug timing in irinotecan pharmacokinetics, in DNA-bound TOP1 amount during SN38 exposure, and in drug-induced apoptosis. Clock silencing through siBMAL1 exposure ablated all the chronopharmacology mechanisms, demonstrating they originated from the molecular circadian clock. The PK-PD model developed in the first study was fitted to these new datasets and achieved a good fit. Parameter sensitivity analysis performed on the model allowed to assess the relative importance of pharmacological proteins with respect to irinotecan chronotoxicity. They concluded on the dominant role of the activation enzymes carboxylesterases and the detoxification enzymes UGT1As in the phase and amplitude of the drug chronotoxicity rhythm.

##### b. Irinotecan systems chronopharmacology in mice

As in a multiscale approach, irinotecan chronopharmacology was then investigated in several C57BL/6-based mouse strains, in both male and female mice. Irinotecan toxicity patterns were assessed through mouse survival, body weight loss, and intestinal and hematologic toxicities, and three classes of chronotoxicity were found (Ahowesso et al., 2010; Li et al., 2013).

To gain a mechanistic understanding of irinotecan chronopharmacology, a whole-body model of irinotecan chronoPK-PD was designed, building on the cellular model from the prior in vitro studies (Ballesta et al., 2012). It included seven compartments representing the blood, the liver that plays a critical role in drug metabolism, both main toxicity targets—intestine and bone marrow, the noneliminating tissues, and the tumor to account for drug efficacy. The intestine was divided into two compartments, representing intestinal mucosa cells, and intestinal lumen, respectively. A bidirectional transport was assumed between the blood and all organs, except for the transport from the intestinal cells to the hepatic portal vein, which was represented by a flow directly toward the liver. The enterohepatic circulation was also modeled by a drug transport from the liver to the intestinal lumen, which stood for biliary excretion. Renal and intestinal clearance were included, respectively, in the blood and intestinal lumen compartments. Model parameters were either inferred from the in vitro studies or estimated from experimental data available in the three classes of irinotecan chronotoxicity (Li et al., 2013). Both PK time–concentration profiles in blood and tissues after administration at the best and worst time of tolerability and circadian expression of pharmacological proteins were integrated in parameter estimation (Ballesta et al., 2012). Results showed a good model-to-data fit for male B6D2F1 male mice. This whole-body chronoPK-PD model is being extended to further study the molecular determinants of irinotecan chronopharmacology in other mouse categories.

#### 2. Oxaliplatin

Oxaliplatin is a platinum complex that is effective against human colorectal cancer. The main adverse events of the drug include diarrhea, hematologic suppression, and peripheral sensory neuropathy. Circadian rhythms in oxaliplatin PK, toxicities, and antitumor efficacy have been described in mice and in cancer patients (Lévi et al., 2010). In addition, high Bmal1 expression was recently associated with increased efficacy of oxaliplatin-based chemotherapy against colorectal cancer (Zeng et al., 2014).

Altinok et al. (2009) used a physiologically-based PK model coupled to a cellular automaton of the cell cycle to study oxaliplatin chronotoxicity. The authors modeled the drug action as an increase in the propensity of quitting the cell cycle, regardless of the cell current phase as oxaliplatin cytotoxicity is not cell cycle phase-specific. A chronoPK model was designed accounting for oxaliplatin transport between plasma and cells, drug binding to plasma proteins, and intracellular detoxification, although reduced reduced glutathione. Both plasma protein and reduced glutathione amounts were assumed to display circadian variations, and their respective acrophases were set to 4:00 PM and 12:00 PM according to clinical data and extrapolated results from mouse investigations. Oxaliplatin cytotoxicity was predicted to be more pronounced when delivered at 4:00 AM rather than at 4:00 PM as a consequence of lower protein binding and detoxification. This theoretical result aligned with clinical findings on oxaliplatin chronotoxicity (Lévi et al., 2010).

Another work undertook a different mathematical approach to study time-scheduled regimens of oxaliplatin administered to mice bearing Glasgow Osteosarcoma (Basdevant et al., 2005). The model represented oxaliplatin toxicity in two cell populations: 1) a population of tumor cells and 2) a population of fast renewing healthy cells in the jejunal mucosa. First-order pharmacokinetics was assumed for total platinum concentration in the plasma, the jejunal mucosa, and the tumor compartments. The healthy cell population was modeled by two ordinary differential equations representing mature and young cells, the latter ones being sensitive to oxaliplatin. The tumor dynamics was assumed to follow a Gompertz law modified to account for oxaliplatin toxicity. Circadian rhythms were assumed for oxaliplatin cytotoxicity against young enterocytes and tumor cells. The model was partly calibrated to literature data and used in optimal control computations. Therapeutic strategy consisted in maximizing drug effect on cancer cells under the constraints of healthy cells remaining above a given threshold representing acceptable toxicity. Optimal infusion profiles were not superimposable onto the 24-hour cosine wave of oxaliplatin delivery currently used in the clinics. Yet, the authors concluded that more work was needed on parameter estimation and model validation before taking these results to the clinics.

#### 3. 5-Fluorouracil

The 5-FU is an antimetabolite drug that has been administered to colorectal cancer patients since 1957 and still remains the cornerstone of current therapeutic strategies against intestinal malignancies. Large circadian variations modulated 5-FU tolerance and efficacy both in mice and in cancer patients (Lévi et al., 2010).

The 5-FU chronotolerance was studied through an agent-based model representing healthy and tumor human cells (Altinok et al., 2009). Cell cycle phase duration and circadian timing were inferred from the literature for healthy cells and were varied for cancer cells as a result of circadian disruption. Because the drug has a short half-life of approximately 10–20 minutes, no PK model was considered and drug exposure of the tissues was assumed to be similar to administration profiles. The 5-FU cytotoxicity was represented as an increase in the probability of cells in S-phase to exit the cell cycle at the G2/M checkpoint. In this model, 5-FU chronotolerance was driven by the proportion of cells in S-phase and was lower for drug administration at 4:00 AM for a cell cycle of 24 hours. The authors further investigated the effect of intercellular variability in cell cycle phase durations. Maximum 5-FU efficacy obtained for optimal circadian time of administration increased with cell cycle desynchronization within a cell population because the fraction of cells in S-phase was always larger than at the optimal time in well-synchronized populations. This theoretical finding provided a theoretical rationale, which further supported chronotherapeutics, as tumor tissues often escape from the circadian control responsible for synchronization.

Another work aimed to develop a physiologically-based model of 5-FU PK-PD and their circadian control in humans (Lévi et al., 2010). The model represents 5-FU plasma administration and further degradation by hepatic dihydropyrimidine dehydrogenase, the drug cellular uptake and active efflux (ABCC11), intracellular metabolism into the active metabolite fluorodeoxyuridine monophosphate (FdUMP) through thymidine kinase (TK), and reversible binding of FdUMP to thymidylate synthase (TS). The coadministration of leucovorin is represented through its intracellular active compound methylene tetrahydrofolate, which stabilizes FdUMP-TS complexes into irreversible ternary complexes. Drug-induced overexpression of efflux pumps is also represented through the activation of a generic nuclear factor enhancing ABC transporter expression. Circadian rhythms are assumed in dihydropyrimidine dehydrogenase and TS protein activities.

### C. Toward Personalized Chronotherapeutics: from Biomarkers to Personalized Chronomodulated Infusions

Modeling anticancer drug chronoPK-PD and action on cell populations or tissues constitutes the first preclinical step toward the design of clinically-relevant physiologically-based models. Indeed, mouse-to-human scaling methods already exist and are undergoing further development, to help design human models based on validated in vivo–in silico circadian studies. Sensitivity analysis performed on such generic clinical models informs on the key determinants of anticancer drug chronotoxicity and chronoefficacy to be further investigated to obtain patient-specific chronoPK-PD models. Hence, such modeling approach can integrate the continuous recording of key circadian parameters and tumor-related markers to account for CTS disturbance and disease evolution in therapeutics optimization for a given patient. Those continuous circadian individual datasets combine to patient general information (age, sex, lifestyle habits, chronotype, concomitant medications...), clock and pharmacology gene polymorphisms, cancer stage, phenotype, and molecular characteristics. Such multidimensional features can be integrated into dedicated physiologically-based models enabling patient-tailored anticancer chronotherapy computation on a real-time basis.

Thus, the quest for personalized chronotherapeutics has favored the development of Domomedicine platforms, allowing for the continuous monitoring of circadian and disease-specific markers in nonhospitalized patients. A first successful conceptual, technological, and clinical investigation was conducted within the European Project InCASA (http://www.incasa-project.eu) in 31 metastatic cancer patients on treatment (Innominato et al., 2016a). Self-measured body weight, self-rated symptoms using the M.D. Anderson Symptom Inventory, and circadian rest-activity rhythm recording with a wrist-accelerometer (actigraph) were transmitted daily by patients to a server via the Internet, using a dedicated platform installed at home, over an average duration of 58 days. The French State supported PiCADo project and then developed a mobile multiuser and multipathology telecommunicating platform, fit for the monitoring of individual patients’ parameters at home (Maurice et al., 2015). The platform integrates several lightweight and portable technologies made interoperable (sensor, collector, geolocation watch, digital tablet, digital pen collector, information systems, electronic health records), to allow noninvasive and automatic collection of different markers of biologic rhythms (activity, position, temperature) and health status of the patient (body weight, self-rated symptoms, and quality of life, etc.) at home or during his or her daily activities. Authorized users can access record information via a secure web interface, add different type of patient information (health, nutrition, psychology, etc.), and communicate with other caregivers via the same interface. Automatic preanalysis of data is coupled to notifications sending to care professionals, which they can reset. According to the data, professional caregivers can also propose adapted dietician or psychologic support services to their patients. The PiCADo project has also allowed the design of the PiCADomo clinical study, which aims to establish the first multidimensional database on the health relevance of circadian rhythms, measured in real-life condition in people receiving complex circadian chemotherapy outside the hospital.

## VI. Systems Chronotherapeutics for Other Pathologies

In this study, we review published mathematical works aiming at improving pharmacotherapies of immunologic, inflammatory, cardiovascular, and metabolic diseases. There has been no chronotherapeutics modeling for psychiatric diseases to the best of our knowledge, despite bipolar and other mental disorders having inspired dynamical modeling or chaos theory applications, resulting in original physiopathological perspectives (Goldbeter, 2013; Kupfer et al., 2015).

### A. Rheumatology and Immunity

Tight links exist between the CTS and the immune system, as multiple immunologic processes, such as susceptibility to infection, immune cell recruitment, or systemic proinflammatory cytokine levels, are under circadian control (Geiger et al., 2015; Labrecque and Cermakian, 2015). Daily variations in the intensity of symptoms of diseases involving the immune system have also been reported, as in rheumatoid arthritis and osteoarthritis (Geiger et al., 2015; Labrecque and Cermakian, 2015). Anti-inflammatory medications represent a main therapeutic class to combat those rheumatologic diseases, and the circadian drug timing plays a crucial role for their pharmacokinetics, tolerability, and efficacy. For instance, evening dosing resulted in both lowest Cmax or Cmax/Tmax (absorption estimate) and least toxicities for nonsteroidal anti-inflammatory drug indomethacin or ketoprofen both in healthy subjects and in osteoarthritic patients (Clench et al., 1981; Guissou et al., 1983; Lévi et al., 1985; Ollagnier et al., 1987). These findings led to an investigation of the clinical relevance of dosing time for an oral sustained-release form of 75 mg indomethacin (Chrono IndocinR) in patients with osteoarthritis of hip or knee. Overall, 517 patients participated in one of four randomized multicentre crossover trials, including a placebo-controlled double-blind study. Each patient took a single oral dose of indomethacin sustained release in the morning, at noon, and in the evening for 1 week each. Adverse events and pain control were the main endpoints. The incidence of gastrointestinal or central nervous system–related adverse events was nearly fivefold as large after morning as compared with evening dosing in each trial (35 versus 7%). As a result, the rate of toxicity-related treatment withdrawals was threefold as high following morning as compared with evening indomethacin sustained release intake. In contrast, optimal pain control varied according to the daily pain pattern. The patients with a typical mechanical pain predominating in the early evening benefitted most from morning or noon intake. In contrast, those patients whose pain had an inflammatory component, as revealed by an early morning exacerbation or an arrhythmic profile, benefitted most from evening dosing (Lévi et al., 1985; Reinberg and Lévi, 1987). The results emphasized the need to consider pain dynamics, as a disease-related circadian biomarker, to jointly optimize tolerability and efficacy in individual patients.

Rheumatoid arthritis is an inflammatory disease associated with joint pain and stiffness. Disease symptoms display circadian rhythms as they tend to be more severe in the morning (Buttgereit et al., 2015). Such variations have been correlated in rheumatoid arthritis patients with overnight increases in the systemic levels of proinflammatory cytokines that usually peak in the early morning. This finding led to the development of several efficient modified-release prednisone formulations achieving circadian drug exposure starting near the middle of the night span, after an administration at bedtime (Buttgereit et al., 2015). Indeed, a multicentre double-blind randomized clinical trial involving 288 rheumatoid arthritis patients confirmed a better pain and stiffness control of chrono-released prednisone as compared with the conventional morning prednisone, with statistical significance (Buttgereit et al., 2008). Other disease-modifying drugs such as methotrexate were also shown to be better tolerated and more effective in patients, following evening dosing (To et al., 2011).

The molecular mechanisms underlying rheumatoid arthritis symptoms were amenable to mathematical modeling based on the circadian control of key components of the neuroendocrine–immune system (Meyer-Hermann et al., 2009). The model represents the interplay between cortisol, noradrenaline, and tumor necrosis factor (TNF)-*α* dynamics at the whole-body level. Theoretical therapeutic predictions supported best efficacy of glucocorticoids regarding inhibition of TNF-*α* secretion following their delivery between midnight and 2:00 AM, that is, at a time when this cytokine started being released in the circulation. Interestingly, this optimal timing corresponded to a drug administration during the early increase of TNF-*α* plasma level rather than around its peak value.

Rheumatoid arthritis is under circadian control, but the disease can also disrupt the CTS. In a mouse model of arthritis, both the circadian rhythms in PER2 protein in the synovial cells of foot joints and the clock gene transcription patterns in the spleen were strongly altered as compared with healthy mice (Labrecque and Cermakian, 2015). Interactions between the CTS and cartilages occur through multiple molecular processes both at the systemic and local levels in healthy conditions (Buttgereit et al., 2015; Yang and Meng, 2016). Anti-inflammatory treatments can also profoundly alter the CTS as a function of dose and timing. Indeed, the delivery of glucocorticoids at early night suppressed the endogenous cortisol secretion, resulting in functional adrenal insufficiency. The intake of a single dose of indomethacin ablated most cognition, performance, or physiology rhythms following morning intake, whereas 10 of 11 measured rhythms were maintained following evening dosing (Clench et al., 1981). Taken together, the personalization of rheumatologic disease management represents a clinical challenge involving the interplay of several physiologic systems and their chronopharmacological control. Such complex multiscale problem may greatly benefit from systems approaches integrating patient-specific features of both diseased tissues and drug chronoPK-PD using the same modeling approach developed for cancer (reviewed in *Cancer as a Driver for Systems Chronotherapeutics*).

### B. Cardiovascular Diseases

Marked circadian rhythms characterize most physiologic, biochemical, and molecular parameters that impact on the cardiovascular system. These include heart rate; blood pressure; peripheral vascular resistance; blood volume; circulating and intracellular concentrations of potassium, sodium, and other ions; and circulating plasma levels of proteins and albumin, as well as clock genes and clock-controlled genes and proteins in heart and arteries (Portaluppi and Hermida, 2007; Smolensky et al., 2016). Such circadian organization translates into predictable daily changes in the occurrence of many cardiac events. For instance, most hypertension bouts, cardiac arrhythmias, myocardial infarction, or sudden cardiac death happen in the morning in the general population (Portaluppi and Hermida, 2007; Smolensky et al., 2016). However, we are lacking a mechanistic understanding of the CTS alterations that contribute to cardiovascular pathologies. Statistical and mathematical modeling approaches have started addressing this challenge. Fijorek et al. (2013) developed a model investigating the influence of calcium, potassium, and sodium circadian rhythms on the electrocardiogram and resulting interval between the start of the Q wave and the end of the T wave in the heart’s electrical signal (QT). They concluded that potassium daily variations had the most significant effect on QT circadian rhythms compared with sodium and calcium, thus advocating for its choice as a potential circadian biomarker of the cardiovascular system.

The efficacy of several medications against cardiovascular diseases varied largely according to the circadian time of administration (Portaluppi and Hermida, 2007; Smolensky et al., 2015a), although differences between subgroups of patients receiving morning or bedtime drug administration have not been universally observed (Stranges et al., 2015). Indeed, most drugs against hypertension achieved both greater improvement of blood pressure circadian profile and lower risks of cardiovascular events, following their oral intake at bedtime compared with morning administration (Smolensky et al., 2016). Several physico-chemical studies further developed modified release formulation of valsartan to achieve late night/early morning exposure after a bedtime administration (Kshirsagar et al., 2011; Biswas and Kuotsu, 2017). Classic PK-PD models have been developed for cardiovascular medications linking drug dose or plasma concentration to their effect on the QT interval in an empirical manner, and modeling efforts have been made to extend them to account for the circadian control of the drug effect (Piotrovsky, 2005; Huh and Hutmacher, 2015). More recently, mixed-effect models were further used to predict the drug-induced QT prolongation, and modeling reliability was improved when integrating daytime variations (Huh and Hutmacher, 2015). A similar approach was used to study the effect of three compounds on QRS and PR intervals in dogs (Bergenholm et al., 2016). Interestingly, taking into account circadian rhythms improved the fit for the PR model, but not for QRS modeling. Such empirical models provide hints for the design of more detailed mechanistic ones. The subsequent development of a systems approach to the chronotherapeutics of cardiovascular diseases should expectedly help design personalized circadian administration algorithms.

### C. Metabolic Diseases, Diabetes, Obesity

Energy metabolism is under the control of the CTS at multiple scales, as both systemic factors driven by the SCN and cellular clocks regulate key metabolic processes (Sukumaran et al., 2010; Oster et al., 2016). Many disorders in glucose regulation, such as diabetes (Qian and Scheer, 2016) or obesity (Laermans and Depoortere, 2016), and their pharmacotherapies are also under robust circadian control. Recently, systems approaches have aimed at studying the dynamics of the circadian processes possibly impacting on energy metabolism. Because white adipose tissue plays a critical part in many metabolic disorders, Sukumaran et al. (2011) developed a mechanistic model of the regulation by the CTS of adipokine expression in those tissues, also incorporating the glucose/free fatty acid/insulin system and the activity of methylprednisolone in rats. Circadian oscillations were considered in the transcription of glucocorticoid receptors, leptin, and adiponectin in the tissues and in the plasma levels of glucose, free fatty acid, insulin, leptin, and adiponectin. These quantities were fitted to experimental results in rats showing robust circadian rhythms except for adiponectin (Sukumaran et al., 2011). Such systems approach gave insights into the molecular mechanisms of methylprednisone chronopharmacology driving the modification of glucose, free fatty acid, and insulin plasma circadian profiles after drug administration. The model also predicted circadian oscillations in those plasma quantities that were not captured in the experiments due to the small number of sampling times. Regarding diabetes, portable pumps or implanted artificial pancreas are now available to supply insulin along preprogrammed patterns (Russell, 2015; Visentin et al., 2015). Both modeling and statistical efforts are being made to optimize the insulin administration scheme according to the patient’s sex, age, and corresponding biologic rhythms (Klonoff, 2010; Holterhus et al., 2013; Emami et al., 2016).

## VII. CTS Disruption

Both the disease itself and its treatments can contribute to the disruption of the CTS in patients. We review in this work the incidence of circadian disruption and its impact on treatment outcomes and patient well-being and suggest systems approaches to better understand the pathophysiological mechanisms leading to the desynchronization of circadian functions. We further review existing behavioral and pharmacological strategies to strengthen the CTS rhythmicity and coordination, and available mathematical and statistical tools to personalize them.

### A. Disease-Driven CTS Disruption

Chronic diseases may alter the synchronicity of the CTS by perturbing either the central pacemaker in the SCN and the physiologic messengers it produces toward the body, or directly the rhythms of gene expression in the peripheral organs. Tumor tissues may present disrupted circadian organization due to genetic or epigenetic alterations of clock gene expression, leading to the loss of functional cellular clocks at the single-cell level and/or the desynchrony of cells within a tissue (Savvidis and Koutsilieris, 2012; Ortiz-Tudela et al., 2013). Similar circadian alterations have been described in joint tissues of an animal model of rheumatoid arthritis (Labrecque and Cermakian, 2015).

At the whole-body level, the modulation of small proteins constitutively expressed in the SCN, including ligands of the epidermal growth factor receptor (EGFR)-1 and -3 receptor family, such as tumor growth factor-*α*, epidermal growth factor, and neuregulin-1, respectively, produced inhibitory actions on circadian behaviors in laboratory animals (Kramer et al., 2001, 2005; Snodgrass-Belt et al., 2005). Prokineticin-2 (a form of vascular endothelial growth factor) and cardiotrophin-like cytokine (an interleukin-6–like molecule) administration abolished rhythmic locomotor activity and other circadian behaviors that were restored when these infusions were stopped, supporting the ligand/receptor hypothesis (Cheng et al., 2002; Kraves and Weitz, 2006). Moreover, high-throughput transcriptomic and metabolomic studies in mice bearing lung adenocarcinoma xenografts demonstrated that the tumor modified the circadian rhythms of hepatic metabolism through proinflammatory response via the signal transducer and activator of transcription 3–suppressor of cytokine signaling 3 pathway, this control being independent of the hepatocyte molecular clocks that were not affected by the presence of the malignant cells (Masri et al., 2016).

Furthermore, elevated serum levels of EGFR ligands and proinflammatory cytokines (i.e., interleukin-6 and TNF-*α*) were associated with behavior changes in cancer patients (Dantzer et al., 2008; Miller et al., 2008; Reyes-Gibby et al., 2008). In patients with mCRC, we reported a significant association of fatigue and appetite loss in those with higher levels of tumor growth factor-*α*, an observation that is consistent with the preclinical model of hypothalamic modulation of circadian behavior related to the EGFR family (Rich et al., 2005). EGFR tyrosine kinase inhibitors are associated with a rapid improvement of cancer patients’ symptoms of well-being and appetite that are consistent with this model (Natale, 2004; Bezjak et al., 2006). These observations were prospectively tested in a small study on the association of normalized rest-activity patterns and symptomatic improvement in nonsmall cell lung cancer patients receiving gefitinib (Iurisci et al., 2007). Although elevated proinflammatory cytokine levels account for about half of cancer patients with altered circadian function, other mechanisms are most likely involved, including drug-induced CTS disruption, blunted synchronizers from disrupted feeding routine, minimal and untimely light exposure, or circadian alterations mediated by other brain areas. Hence, systems medicine approaches encompassing all factors toward CTS strengthening would not limit their usefulness to cancer, but could be applied to other chronic conditions, such as joint (Berenbaum and Meng, 2016), renal (Koch et al., 2009), liver (Tahara and Shibata, 2016), cardiovascular (Portaluppi et al., 2012; Smolensky et al., 2015c), metabolic (Asher and Schibler, 2011; Bass, 2012), neurologic (Smolensky et al., 2015b; Videnovic and Zee, 2015), and psychiatric (Wulff et al., 2010; McClung, 2013) diseases. Indeed, altered circadian function has been described in several diseases of the aforementioned systems, and their function has been shown to be affected by circadian disruption in otherwise healthy subjects (Reddy and O'Neill, 2010; Roenneberg and Merrow, 2016).

### B. Drug-Induced CTS Disruption

Some medications can be profoundly disruptive to circadian cycles, including anticancer drugs and agents used against inflammatory, autoimmune, or metabolic diseases (Hrushesky et al., 2009; Maier et al., 2009; Zhang et al., 2009a; Innominato et al., 2014; Roenneberg and Merrow, 2016). Indeed, a large number of therapeutic molecules have an impact on the CTS either through direct intracellular activity in the SCN or in the peripheral tissues or through interference with SCN-induced physiologic signaling. Intracellular drug control implies either direct interference with the molecular clock such as lithium (Hirota et al., 2012), or indirect through changes in cellular pathways that interact with the clock (e.g., double-strand breaks, glucose metabolism). Supportive care drugs, especially centrally-acting medicines, can affect circadian functions given the multiple neuromediator receptors expressed by human SCN neurons (Sprouse, 2004).

However, very few other medications have adverse effects as toxic as anticancer agents (Dantzer et al., 2012; Di Maio et al., 2016). In murine models, 12 anticancer medications have shown to affect the circadian functions, depending on both the drug dose and timing (Lévi et al., 2010). For all considered drugs, the dosing time of minimum circadian disruption corresponded to that of minimum toxicities on healthy tissues. In addition, mice treated with immunotherapy of interferon (IFN)-*α* displayed an altered circadian rhythm of locomotor activity, core body temperature, and clock gene expression levels in both the SCN and the periphery (Ohdo et al., 2001; Koyanagi and Ohdo, 2002). The link between immunotherapy-mediated CTS disturbance and the resulting effects on the host is intriguing, considering that early neurobehavioral symptoms, including depressive, neurovegetative, and somatic (namely, fatigue, anorexia, and sleep disturbances) complaints, have been documented in cancer patients receiving immunotherapy with IFN-*α* (Capuron et al., 2000, 2002). Furthermore, baseline-specific vulnerability factors for the development of IFN-induced symptoms included both pre-existing sleep disturbances and exaggerated hypothalamic pituitary adrenal axis response to the initial IFN challenge (Capuron et al., 2003, 2004). In our experience (Ortiz-Tudela et al., 2014, 2016; Scully et al., 2011; Roche et al., 2014), chemotherapy administration in humans does not necessarily induce alteration of circadian rest-activity and/or skin temperature rhythms.

### C. Clinical Impact of CTS Disruption

The relevance of circadian function in health preservation is endorsed by the negative clinical impact of circadian disruption in several illnesses. We will focus in this work on cancer, where the largest experience exists. There are consistent abnormalities of circadian function associated with cancer and its progression, which have been reported in patients (Mormont and Lévi, 1997). For instance, loss of normal diurnal cortisol patterns, which is associated with more awakenings during the night, predicts early mortality with metastatic breast cancer, independent of other prognostic factors (Sephton et al., 2000). Such cortisol pattern alteration also bears independent negative prognostic repercussion in renal, ovarian, and nonsmall cell lung cancers (Cohen et al., 2012; Sephton et al., 2013; Schrepf et al., 2015). Thus, persistently elevated or relatively invariant levels of cortisol may, in turn, stimulate tumor proliferation via differential gluconeogenesis in normal and tumor tissues, activation of hormone receptors in the tumor, or immunosuppression (Sapolsky and Donnelly, 1985; Ben-Eliyahu et al., 1991; Sephton and Spiegel, 2003; Asher and Sassone-Corsi, 2015; Longo and Panda, 2016).

**Fig. 9.**
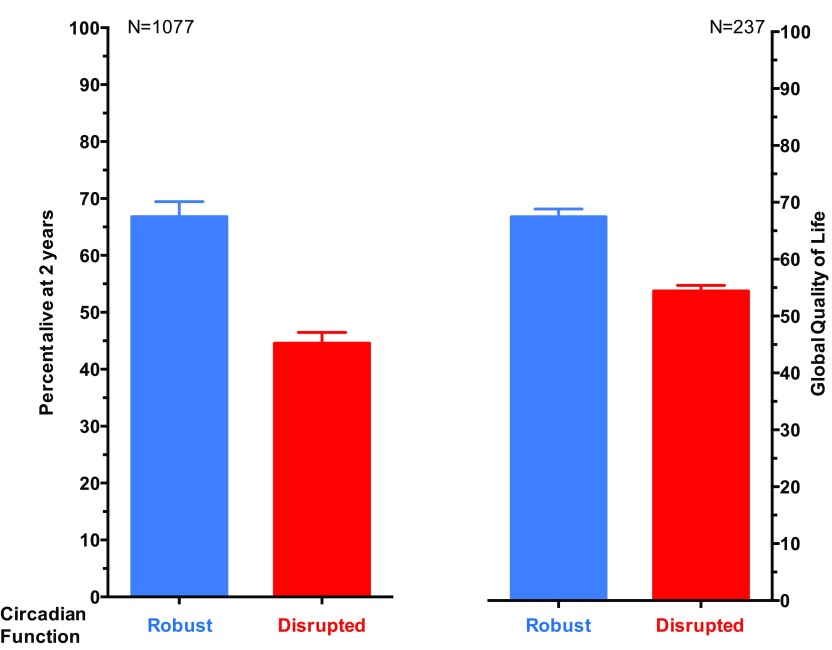
Main outcomes of cancer patients according to circadian functioning status, estimated through assessment of rest-activity or salivary marker rhythms. Left panel: estimated 2-year survival rate (mean + 95% confidence limit) in a total of 1077 cancer patients (Cohen et al., 2012; Innominato et al., 2012; Lévi et al., 2014; Schrepf et al., 2015; Sephton et al., 2013; Sephton et al., 2000). Right panel: global quality of life domain (mean + standard error of the mean), derived from the EORTC QLQ-C30 questionnaire, completed by a total of 237 patients with mCRC (Innominato et al., 2009b).

Similarly, altered rest-activity circadian rhythm assessed either before or during chemotherapy through wrist-actigraphy was associated with worse prognosis in patients with mCRC (Fig. 9) (Mormont et al., 2000; Innominato et al., 2009a, 2012; Chang and Lin, 2014; Lévi et al., 2014). Moreover, in clinical studies involving patients with diverse cancer types and stages whose rest-activity rhythm had been measured by objective parameters derived from wrist-actigraphy, CTS dysfunction was shown to be correlated with subjectively measured worse multidimensional symptoms and health-related quality of life by the patient, as well as with poorer performance status by the physician (Mormont et al., 2000; Mormont and Waterhouse, 2002; Levin et al., 2005; Innominato et al., 2009a,b; Grutsch et al., 2011; Su et al., 2015).

Very closely intermingled with activity and rest is the circadian cycle of wakefulness and sleep (Palesh et al., 2012). Thus, poor sleep efficiency, evaluated with wrist-actigraphy, has been shown to predict shorter overall survival in women with advanced breast cancer (Palesh et al., 2014). Moreover, mCRC patients complaining of subjective sleep trouble displayed abbreviated overall survival too (Innominato et al., 2015), thus supporting the clinical relevance of proper sleep in cancer patients (Palesh et al., 2008; Spiegel, 2008; Ancoli-Israel, 2009; Innominato et al., 2010; Ortiz-Tudela, 2015) and in the general population (Cappuccio et al., 2010). Nonetheless, sleep is not the only patient-reported outcome measurement showing an independent prognostic effect in cancer (Efficace et al., 2006, 2008, 2015; Gotay et al., 2008; Quinten et al., 2009, 2011, 2014; Zikos et al., 2015). Intriguingly, several of these subjective prognostic measures reflect functions with relevant synchronizing influence on the CTS, such as social life, physical activity, or meals (Innominato et al., 2014). Thus, circadian deregulation, endocrine stress response, and immune mechanisms seem to create an ensemble of biobehavioral factors that profoundly impact the tumor biology at multiple interlocking levels (Sephton and Spiegel, 2003; Antoni et al., 2006; Eismann et al., 2010).

Subjects suffering from jet lag or intolerance to shift work might experience fatigue, insomnia or hypersomnia, anxiety, depression, distress, irritability, poor ability to concentrate, reduced vigilance, poor performance, both physical and mental, appetite loss, and dyspepsia (Waterhouse et al., 1997; Drake et al., 2004; Reid and Zee, 2004; Foster and Wulff, 2005; Waterhouse et al., 2007). These same symptoms are often experienced by cancer patients along the course of their disease, as a consequence of both the tumor itself and anticancer chemotherapy, immunotherapy, radiotherapy, or surgery (Cleeland et al., 2000; Walsh et al., 2000; Teunissen et al., 2007). Furthermore, cancer patients are more likely to experience several symptoms at the same time compared with healthy subjects under circadian perturbations (Dodd et al., 2004; Chen and Tseng, 2006; Walsh and Rybicki, 2006; Chen and Lin, 2007). Statistical analysis allowed the identification of clusters of three or more symptoms often co-occurring in patients (Miaskowski et al., 2004; Barsevick et al., 2006; Dong et al., 2014, 2016; Aktas et al., 2016). The main clinical utility of a better understanding of the patterns of association, interaction, synergy, etiology, and pathophysiology of concomitant symptoms producing specific clinical outcomes (both in terms of prognosis and of patient-reported functional outcomes) derives from the possibility of multimodal therapeutic interventions aimed at relieving the clustered symptoms. Several studies have explored the prevalence, severity, and distress of symptoms in cancer patients to better define symptom clusters sharing a common pathophysiology (Cleeland et al., 2000; Chen and Tseng, 2006; Walsh and Rybicki, 2006; Chen and Lin, 2007; Dong et al., 2014). Despite the different symptom assessment techniques, the various statistical methods, and the heterogeneous patient cohorts, it has been found by independent groups that fatigue, drowsiness, poor sleep, and lack of appetite, together with anxiety and depression, tend to show a stronger relationship among them than with other symptoms (Cleeland et al., 2000; Chen and Tseng, 2006; Walsh and Rybicki, 2006; Chen and Lin, 2007; Rich, 2007; Innominato et al., 2009b; Aktas, 2013; Berger et al., 2013). Hence, circadian disruption could be perceived by the patients with the complaint of this symptom cluster, and could be a function to be monitored and targeted to relieve them.

### D. Systems Approaches To Study Disease and Drug-Induced CTS Disruption

The precise molecular mechanisms accounting for the observed effects of diseases and medications on CTS functions still need to be elucidated. The fact that medical therapies can elicit the same toxic symptoms and physiology disturbances induced by the neoplasm itself ushers to the hypothesis that common pathophysiological processes may be involved. Thus, altered circadian function engenders modifications in other physiologic functions, including behavioral changes that lead to blunted synchronizing cues, hence creating a vicious circle where the CTS is further exposed to weaker or ill-timed environmental signals. Hence, CTS disruption needs to be taken into account in the search for optimal chronotherapeutics regimens. Molecular mechanisms by which disease and drug exposure influence all components of the CTS is still a current topic for mechanistic investigations, and the complexity and multilevel nature of this challenge advocate for the use of modeling approaches. Recently, a mathematical model has been developed linking, at the molecular level, the circadian clock and its tuning by metabolic cycles, particularly feeding and fasting behaviors (Woller et al., 2016). The issue of diet is notably relevant in cancer chronotherapeutics, for its impact on the response to anticancer treatment (Bass, 2012; Lee et al., 2012; Longo and Mattson, 2014; Longo and Panda, 2016; Sullivan et al., 2016; Vernieri et al., 2016), among other effects.

Furthermore, a systems biology study investigated at the molecular level the reasons that DNA damage arising from ionizing radiation predominantly induces phase advances of the circadian clock in synchronized cell culture, whereas dexamethasone exposure usually leads to both phase advances and phase delays (Hong et al., 2009). To understand the underlying mechanisms of this nonintuitive result, Hong et al. (2009) explored several mathematical models representing the intracellular pathways activated by DNA damage in cultured cells synchronized with dexamethasone. They concluded that radiation-induced DNA damage activates the CHK2 kinase that phosphorylates and degrades unbound PER proteins, but does not interact with PER in complex with BMAL1/CLOCK dimers. The clock is thus advanced by two molecular mechanisms, as follows: 1) unbound PERs are prematurely degraded, and 2) BMAL1/CLOCK continues to be repressed by PER. Next, the authors studied the autocatalytic positive feedback loop arising from the fact that the degradation rate of PER monomers was assumed to be greater than that of PER as dimers or in complexes with other proteins in the models. Thus, PER stabilizes itself by forming complexes that form the basis of an autocatalytic process. Including this positive feedback mechanism in the model was in fact mandatory to reproduce the experimentally observed predominance of phase advances.

### E. Systems Approaches for Coping Strategies: the Clock as a Target

**Fig. 10.**
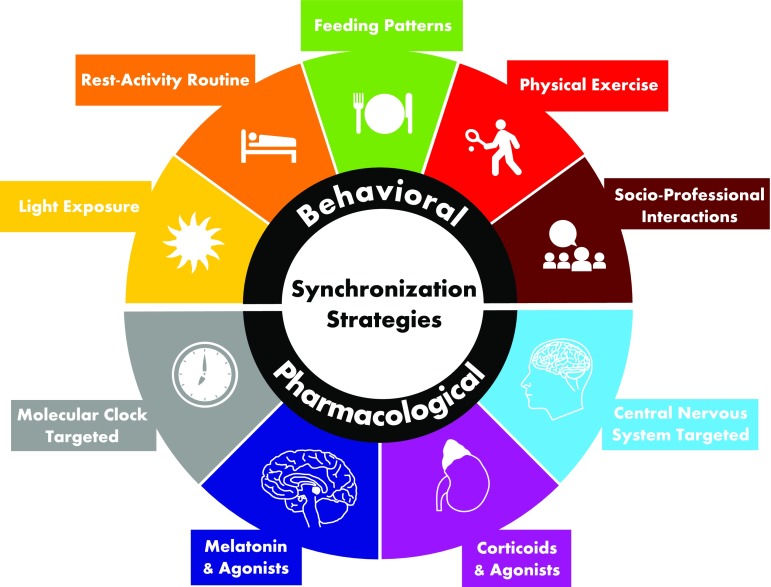
Available behavioral and pharmacological strategies to restore circadian rhythms in patients. The timing and regularity of bright light exposure, physical exercise, social and family life, and sleep-wake routine affect the function of the central clock, whereas the fasting-feeding schedule impacts on metabolism-linked peripheral clocks. Novel synthetic agonists or modified-release formulations of the hormones melatonin (e.g., Ramelteon, Tasimelteon, Agomelanine) and cortisol (e.g., delayed-release prednisone) can be used to target the central clock, as well as the peripheral oscillators in tissues equipped with the specific receptors. Several drugs used to treat psychiatric conditions, thus affecting neuronal function in the central nervous system, such as lithium, selective serotonin-uptake inhibitors, anxiolytic and hypnotic GABA agonists, or novel sleep inducers like the orexin-antagonist Suvorexant, can directly or indirectly modulate the function of the central clock, because SCN neurons are equipped with receptors of these drugs, or receive neuronal input from other brain areas affected by these drug classes. Finally, recent small molecules targeting core proteins of the circadian molecular clock modify their activity and thus impact circadian functions; these include REV-ERB agonist, CK1 inhibitor, SIRT1 agonist, and CRY activator.

Based on the findings reviewed above, one main question arises: are therapeutic interventions targeting the CTS able to improve health-related quality of life of patients, together with treatment efficacy and tolerability? This question is yet unanswered, but interest in this issue is growing and warranted by findings suggesting that stimuli that exert a pacemaker effect on the biologic clock can influence disease progression. Chronotherapeutics may help in strengthening or weakening the entrainment of the biologic clock to the circadian environment cues by timely administering the right stimuli to the patient in need. Behavioral or pharmacological therapies exert different effects on the CTS and subsequently on disease progression in different clinical populations, and sound preclinical research is needed to give the clinicians molecular targets for interventions on the CTS (Fig. 10).

This issue cannot be skipped. The light-dark cycle and many other unavoidable stimuli, such as timing of meals, of physical activity, and of socio-professional interactions, exert a synchronizing effect on the biologic clock. Confirming the classic belief that humans and their environment are inseparable, it is then impossible to avoid exposure to environmental stimuli that act on brain neurotransmitter function and on the transcription of clock genes, thus exerting a pacemaker effect on the CTS (Benedetti et al., 2007). Because most diseases and their treatments are influenced by these stimuli, a strict control of the exposure to Zeitgeber should be recommended in patients.

#### 1. Behavioral Synchronizing Strategies

The easiness to act on the circadian clock machinery via the manipulation of the light-dark and sleep-wake cycles has led to the development of a variety of therapeutic techniques currently used in the treatment of psychiatric conditions, for example, light therapy, dawn simulation, total or partial sleep deprivation, and sleep phase advance (Wirz-Justice et al., 2005). Interestingly, preliminary data indicate a potential benefit of these therapies in cancer as well (Neikrug et al., 2012; Jeste et al., 2013; Redd et al., 2014). These techniques are devoid of serious side effects, and could then be easily translated to other medical conditions.

The design of a theoretical framework is to optimize the sleep and light scheduling toward rapid circadian resynchronization of body functions. First, the control of light exposure on the molecular clock was theoretically investigated in Drosophila Melanogaster (Bagheri et al., 2008). Using a dynamical mathematical model of the molecular circadian clock, the authors determined the most sensitive molecular targets to entrain the clock and concluded that, although light was a strong synchronizer, directly acting on clock gene transcription and mRNA degradation model parameters may be even more effective. Regarding light entrainment, the model predicted that to correct initial phase differences between the subject’s internal time and the environment of 0 to 9 hours (i.e., to induce a phase delay), daylight is most effective at the end of the day. On the opposite, to correct initial phase differences of 0 to −6 hours and thus to induce phase advances, daylight is most effective at the start of the day. Roberts et al. (2016) further investigated the functional contributions of different populations of cellular oscillators for light entrainment in Drosophila whole-brain explants. Both their experimental and theoretical works concluded that strong circadian cellular oscillators support robust overall synchrony in constant darkness, whereas weaker oscillators facilitate light-induced phase shift in the network synchrony through transient cell-cell desynchrony and damped amplitude at the single-cell level.

Of note, in contrast to flies, the mammalian molecular clock is not directly light sensitive (Roberts et al., 2016). Yet, similar results were observed in mouse SCN and lung extracts, showing that lung cells were entrained to various Zeitgeber cycles, whereas SCN neurons were not (Abraham et al., 2010). Mathematical investigations validated through dedicated experiments concluded that strong cell-to-cell network coupling in the SCN explained these tissue-specific entrainment properties rather than cellular differences. Further pluridisciplinary investigations have shown that the neurotransmitter GABA was central in synchronizing circadian rhythms among individual SCN neurons (DeWoskin et al., 2015). Also, several systems biology approaches intended to explain the singularity behavior in which robust circadian oscillations of a cell population can be abolished after a single stimulus, such as a light or temperature pulse, applied at the appropriate timing and intensity. They demonstrated that singularity behavior arises from the loss of synchronization of the cellular oscillators rather than from arrhythmicity of each individual molecular clock (Ukai and Ueda, 2010).

In the context of jet lag arising from travel across time zones, Dean et al. (2009) have proposed a semimechanistic mathematical model of the circadian pacemaker aiming to design sleep- and light-based countermeasures that allow a rapid alignment of the CTS with the new environment schedule. The developed algorithms allowed for the design of optimal behavioral strategies achieving rapid circadian re-entrainment and subsequent improvement of neurobehavioral performance. More recently, the same model was used in an optimal control investigation to optimize light exposure and avoidance for efficiently re-entraining the human CTS (Serkh and Forger, 2014).

Meal timing is also an effective mean to entrain the CTS. In rodents, it has been shown that regularly time-restricted feeding can shift the molecular clockwork in peripheral tissues (Damiola et al., 2000; Challet et al., 2003; Shibata et al., 2010; Sherman et al., 2012; Longo and Panda, 2016). Moreover, in mice exposed to chronic jet lag conditions, restricted feeding time was able to improve CTS function, with increased amplitude of temperature circadian rhythm (Wu et al., 2004). This phenomenon was associated with a partial rescue of the more rapid tumor growth induced by photic functional CTS disruption (Wu et al., 2004). Anorexia is also a prevalent affliction ensued by cancer patients, and those with circadian disruption and other chronic conditions at large (Foster and Wulff, 2005; Innominato et al., 2009b). In cancer, appetite can be increased and the development of cachexia delayed, through the use of a novel ghrelin mimetic anamorelin, with proven orexic effects (Temel et al., 2016). Furthermore, so far preclinical, approaches have been proposed to stimulate appetite and energy intake in cancer cachexia, for example, using melanocortin 4 receptor antagonism (Dallmann et al., 2011; Peter et al., 2013). These pathways could be further exploited to manipulate appetite and promote optimal food intake timing (Shibata et al., 2010; Mattson et al., 2014; Zarrinpar et al., 2016).

#### 2. Pharmacological Synchronizing Strategies

Such behavioral interventions may be supplemented with pharmacological therapies. Although old and novel sleep inducers can improve sleep in selected populations (Rajaratnam et al., 2009; Lemoine and Zisapel, 2012; Kuriyama et al., 2014; Michelson et al., 2014; Lockley et al., 2015; Innominato et al., 2016b; Liu et al., 2016; Wilt et al., 2016), cancer patients, sick people with other conditions, and the general population suffering from sleep troubles seem to benefit from the concomitant use of both hypnotic drugs and cognitive and behavioral therapies (CBT) (Bootzin and Epstein, 2011; Riemann et al., 2011; Langford et al., 2012). Conversely, CBT for insomnia remains the first-choice treatment in this setting, in both cancer patients and subjects with primary insomnia (Trauer et al., 2015; Johnson et al., 2016). Behavioral interventions yield better results than pharmacological ones regarding fatigue management for which encouraging outcomes have been described with contemplative therapy, tai-chi, and yoga interventions (Mustian et al., 2007; Minton et al., 2013; Saligan et al., 2015), whereas psychostimulants have shown limited activity (Carroll et al., 2007; Ruddy et al., 2014).

Psychotherapy and pharmacotherapy can be combined to achieve best results in depression. Thus, alongside antidepressants, CBT, mindfulness, and psychodynamic therapy are routinely used to treat depressive state, either primary or cancer-related (Hirschfeld et al., 1997; Mann, 2005; Ebmeier et al., 2006; Dauchy et al., 2013; Craighead and Dunlop, 2014; Walker et al., 2014). From the circadian perspective, of particular interest is the demonstration of clinical activity in refractory bipolar disorder of the interpersonal and social rhythm therapy (Frank, 2007; Frank et al., 2007). This intervention fully integrates the concept of circadian resynchronization into the management of psychiatric conditions associated with circadian disruption and lacks any significant side effect.

Another phase-resetting approach involves the use of endogenous hormone melatonin, or of its more recent analogs (Redfern et al., 1994; Geoffriau et al., 1998; Skene et al., 1999; Touitou and Bogdan, 2007; Cardinali et al., 2012; Rivara et al., 2015; Liu et al., 2016). A mathematical model of the circadian endogenous production and clearance of melatonin was designed and incorporates light-induced melatonin suppression and circadian phase shift (St Hilaire et al., 2007). It also includes a compartment to model salivary melatonin levels, which is widely used in clinical settings to determine circadian phase of individual subject. This model was recently supplemented to include the PK of oral exogenous melatonin and phase-shifting effects via melatonin receptors in the SCN (Breslow et al., 2013). A more complex physiologically-based model of exogenous melatonin whole-body PK is also available (Peng et al., 2013). These models provide comprehensive tools to optimize melatonin administration and light exposure schedule and can incorporate patient-specific molecular features to personalize the resetting strategy.

Finally, specific targeting of the molecular circadian clock, eliciting different kind and depth of responses, can be obtained using dedicated molecules, including agonists or antagonists of core clock genes (Schroeder and Colwell, 2013). Kim et al. (2013) undertook a systems pharmacology approach to optimize the concomitant administration of the CK1*δ*/*ε* inhibitor PF-670462 and light exposure to manipulate the CTS. They designed a model of the SCN molecular clock incorporating PF-670462 brain and plasma PK and the drug-induced inhibition of CK1*δ*/*ε*. The model predictions that were experimentally validated in mice indicated that chronic CK1*δ*/*ε* inhibition during the first hours of the LD12:12 cycle leads to a stable delay of activity, whereas the same drug given toward the end of the light phase, or with light–darkness schedules involving longer light periods, does not entrain the host clock.

Most of the above-mentioned interventions have solely been tested on their own to treat only a single symptom, and very rarely, maybe with the exception of interpersonal and social rhythm therapy, with the aim of restoring a healthy circadian function. However, given the oftentimes multifactorial pathogenesis of circadian disruption, and the specificity of each patient, at any given point of the clinical course of the disease, for sensitivity to any disrupting factor, it is undeniable that a systems medicine framework is required for an improved care of these systemic symptoms. This approach should integrate both pharmacological manipulations and behavioral changes, with a personalized plan and predictive measures, and include participation of the patients to their well-being. Indeed, this kind of groundwork is advocated for the 5P medicine (Gorini and Pravettoni, 2011; Hood and Friend, 2011; Tian et al., 2012).

## VIII. Conclusions: Expected Benefits and Challenges of Systems Chronotherapeutics

The delivery of medications according to circadian rhythms has shown clinical benefits in randomized trials involving large number of patients with cancer, rheumatologic, cardiovascular, or allergic diseases. However, inter- and intrapatient variabilities have been demonstrated regarding the circadian timing system that governs chronotherapeutics mechanisms. Dedicated systems biology/medicine methodologies enable such challenges to be handled through the integration of patient-specific key parameters within a unique mathematical framework for the design of personalized pharmacotherapies. Systems chronotherapeutics thus represent a conceptual and methodological advance for making chronotherapeutics fit for each individual patient. As such, it will expectedly greatly impact on patients’ health due to the joint improvement in tolerability and efficacy, resulting from the moderation of treatment dynamics by patient- and disease-specific parameters, as well as by therapeutic strategy. This is in sharp contrast with current treatment paradigms, which remain mostly based on empiricism, standardization, snapshot assessments, and reactive decisions. Additionally, systems chronotherapeutics will allow for the reduction of medical complications due to the delivery of even complex treatments on a full outpatient basis. The continuous monitoring of patient well-being and disease in real time, thanks to dedicated interactive Domomedicine platforms, has nowadays entered clinical evaluation for cancer. Indeed, the combination of complex chronotherapeutic administration at home, with multidimensional telemonitoring, and timely behavioral tutoring would support chronic disease patients and their families to enjoy improved daily life, through reduced adverse events, and better efficacy.

Preclinical and clinical physiologically-based systems chronopharmacology approaches have highlighted the need for new quantitative measurements at cell, tissue, and whole organism scales. PK investigations in mice have revealed a crucial role of tissue drug concentrations that highly differ according to circadian timing, whereas plasma PK sometimes showed little differences. Hence, the sole quantification of plasma drug levels does not provide the needed information for chronotherapy optimization. The translation of this finding to the clinics requires the development of noninvasive quantitative imaging techniques. Moreover, physiologically-based models incorporate organ-specific circadian rhythms of protein activities so that precisely evaluating those quantities is crucial for model calibration and subsequent validation. Thus, the development of reliable quantitative techniques measuring the absolute protein levels and activities on a 24-hour basis is needed.

The arising of “omics” technologies may have huge implications for the molecular understanding of the CTS and its interplay with diseases and treatments, and for the implementation of personalized chronotherapy into the clinics. Recent in vitro and in vivo investigations have provided insights into tissue-specific circadian organization through transcriptomic, proteomic, and metabolomic circadian datasets (reviewed in Dallmann et al., 2016; Gumz, 2016). In particular, systems approaches involving high-throughput preclinical studies in the mouse liver have been useful for characterizing the circadian molecular determinants of the clock control on hepatic drug metabolism. Furthermore, circadian “omics” technologies have now been tested in various biologic samples from laboratory animals, such as blood, saliva, urine, and exhaled breath (reviewed in Dallmann et al., 2016). These investigations present a great translational potential as such samples can realistically be collected around the clock in a noninvasive manner in individual patients. Such large-scale datasets could then be combined, through systems methodologies, with other patient data such as rest-activity and temperature rhythmic profiles or genetic polymorphisms toward the prediction of individualized optimal drug timing.

Pharmaceutical and biomedical industries also have great interest in endorsing multidisciplinary systems chronotherapeutic as a cost-effective mean to improve drug development, which currently has a high failure rate (Rosenblatt, 2017). Indeed, physiologically-based mathematical models can be developed to assess patient-specific drug chronoefficacy and chronotolerability from multitype datasets measured through dedicated biomedical devices. Thus, this type of modeling allows for a priori in silico test of therapeutic response of individual patients to a specific drug combination and/or timing, thus providing a critical tool to assist the clinicians’ decision to include a particular patient in a clinical trial. Ultimately, physiologically-based models can be used in optimization procedures to design personalization frameworks taking as inputs multitype datasets in the individual subject and outputting patient-tailored chronomodulated treatments. After its design though preclinical and clinical steps, the personalization algorithm needs to be validated through several clinical stages. As a result, systems chronotherapeutics represent a new methodology for the design of clinical trials, in which each individual patient would receive individualized chronomodulated therapies computed by data-driven mathematical models, a novel approach in need of clinical validation. One of the challenges toward this clinical progression involves the training of clinicians and health professionals, to make them fully understand the grounds and clinical potential of systems medicine approaches. Thus, the European CaSyM project has been emphasizing the need for integrating systems medicine into medical and other medically-related degrees.

## Abbreviations

AIC: 4-amino-5-imidazole-carboxamide
CBT: cognitive and behavioral therapies
CHK: check
CK: casein kinase
CRC: colorectal cancer
CTS: circadian timing system
DDE: delay differential equation
EGFR: epidermal growth factor receptor
FBS: fetal bovine serum
FdUMP: fluorodeoxyuridine monophosphate
5-FU: 5-fluorouracil
IFN: interferon
mCRC: metastatic CRC
MR: modified release
MTIC: 5-(3-methyltriazen-1-yl)imidazole-4-carboxamide
ODE: ordinary differential equation
PD: pharmacodynamics
PDE: partial differential equation
PK: pharmacokinetics
QT: interval between the start of the Q wave and the end of the T wave in the heart’s electrical signal
SCN: suprachiasmatic nuclei
TMZ: temozolomide
TNF: tumor necrosis factor
TOP1: topoisomerase 1
TS: thymidylate synthase
UGT: UDP-glucuronosyltransferase
